# Spatiotemporal single-cell atlas of suture stem cell dynamics in craniosynostosis

**DOI:** 10.1186/s13287-026-04987-6

**Published:** 2026-05-03

**Authors:** Xinyan Chen, Chenzhi Lai, Tian He, Zong Chen, Xiaolei Jin

**Affiliations:** Department of Craniomaxillofacial Surgery, Plastic Surgery Hospital, Chinese Academy of Medical Sciences & Peking Union Medical College, Beijing, 100144 China

**Keywords:** Craniosynostosis, Suture mesenchymal stem cells, Single-cell transcriptomics, Spatial transcriptomics

## Abstract

**Background:**

Craniosynostosis is a congenital disorder characterized by premature suture fusion and aberrant skull morphogenesis. The cellular dynamics and regulatory mechanisms of suture mesenchymal stem cells (SuSCs) in this disease remain poorly defined.

**Methods:**

We integrated single-cell RNA sequencing and 2-μm-resolution Visium HD spatial transcriptomics to build a spatiotemporal atlas of coronal suture cells in *Fgfr2*^*C342Y/*+^ mice, a murine model recapitulating human Crouzon syndrome, alongside wild-type controls across three key developmental stages (E14.5, E18.5, and P3). To obtain near single-cell spatial resolution, we created SpatialCell, which combines morphology-based segmentation and machine-learning classification using a reference trained on our single-cell datasets.

**Results:**

The atlas reveals stage-specific remodeling of SuSC niches and a shift of SuSC spatial associations toward osteogenic mesenchyme in craniosynostosis. Along the SuSC-to-osteoblast trajectory, pre-osteoblasts were depleted earlier than upstream SuSCs, and SuSCs displayed premature acquisition of osteogenic programs near the suture midline. Temporal Gene Ontology patterns indicated early extracellular-matrix disruption, mid-gestation chondrogenic activation, and postnatal mineralization. Network analysis nominated *Foxa3* as a candidate regulator in SuSC subsets; siRNA knockdown of *Foxa3* reduced ex vivo mineralization in the craniosynostosis background. Spatial communication analyses implicated signals from suture meningeal fibroblasts and immune cells that converge on SuSC fate.

**Conclusions:**

Our results support a model where craniosynostosis may involve disrupted temporal coordination of developmental programs, not merely accelerated bone formation. The atlas and analytic framework pinpoint when and where SuSC fate diverges, propose *Foxa3* as an intervention target, and provide a high-resolution resource for mechanistic and therapeutic exploration.

**Supplementary Information:**

The online version contains supplementary material available at 10.1186/s13287-026-04987-6.

## Introduction

Craniosynostosis is a group of craniofacial disorders involving premature fusion of one or more calvarial sutures in infancy, leading to abnormal skull growth, elevated intracranial pressure, and potential neurodevelopmental impairment [1, 2]. Current treatment relies predominantly on surgical intervention, which is frequently followed by resynostosis and the need for reoperation [3]. These limitations underscore the urgent need for effective therapeutic strategies; however, progress remains hindered by limited understanding of the pathogenesis and progression of craniosynostosis.

Previous studies on craniosynostosis have predominantly focused on mature osteoblasts and osteoprogenitors, whereas the role of suture mesenchymal stem cells (SuSCs)—which reside in the suture midline and give rise to osteogenic precursors during normal cranial development—has received comparatively less attention. Emerging evidence indicates that SuSCs are critical for maintaining suture patency and regulating osteogenic homeostasis [4–6]. Notably, SuSCs have been shown to reverse cranial and neurocognitive abnormalities and promote suture regeneration [7]. However, the mechanisms underlying SuSC fate determination, cellular heterogeneity, and niche regulation in disease contexts remain poorly understood.

Although single-cell and spatial transcriptomic technologies have been applied to various skeletal tissues [4, 8–11], comprehensive analyses specifically focused on SuSCs within the cranial suture microenvironment—particularly under disease conditions—remain scarce. Consequently, it remains difficult to determine whether altered lineage specification, aberrant signaling responses, or disrupted spatial organization of SuSCs contributes to premature suture fusion.

In this study, we sought to characterize the spatiotemporal dynamics of SuSCs during normal and pathological suture development by combining single-cell RNA-seq and 2 μm-resolution Visium HD spatial transcriptomics in a gain-of-function *Fgfr2*^*C342Y/*+^ mouse model, originally described by Eswarakumar et al. and widely used as a model of human Crouzon syndrome [12, 13]. These efforts enabled the construction of the cross-genotype, multi-timepoint spatiotemporal atlas of SuSCs, supported by a pipeline that integrates morphology-based segmentation with machine-learning annotation to achieve single-cell-level spatial mapping. By comparing the molecular profiles and lineage trajectories of SuSCs across developmental stages and genotypes, we aimed to pinpoint when and where SuSC fate is diverted in craniosynostosis and to nominate candidate regulatory factors for targeted intervention.

## Results

### A spatiotemporal map of coronal suture development in craniosynostosis

**Fig. 1:**
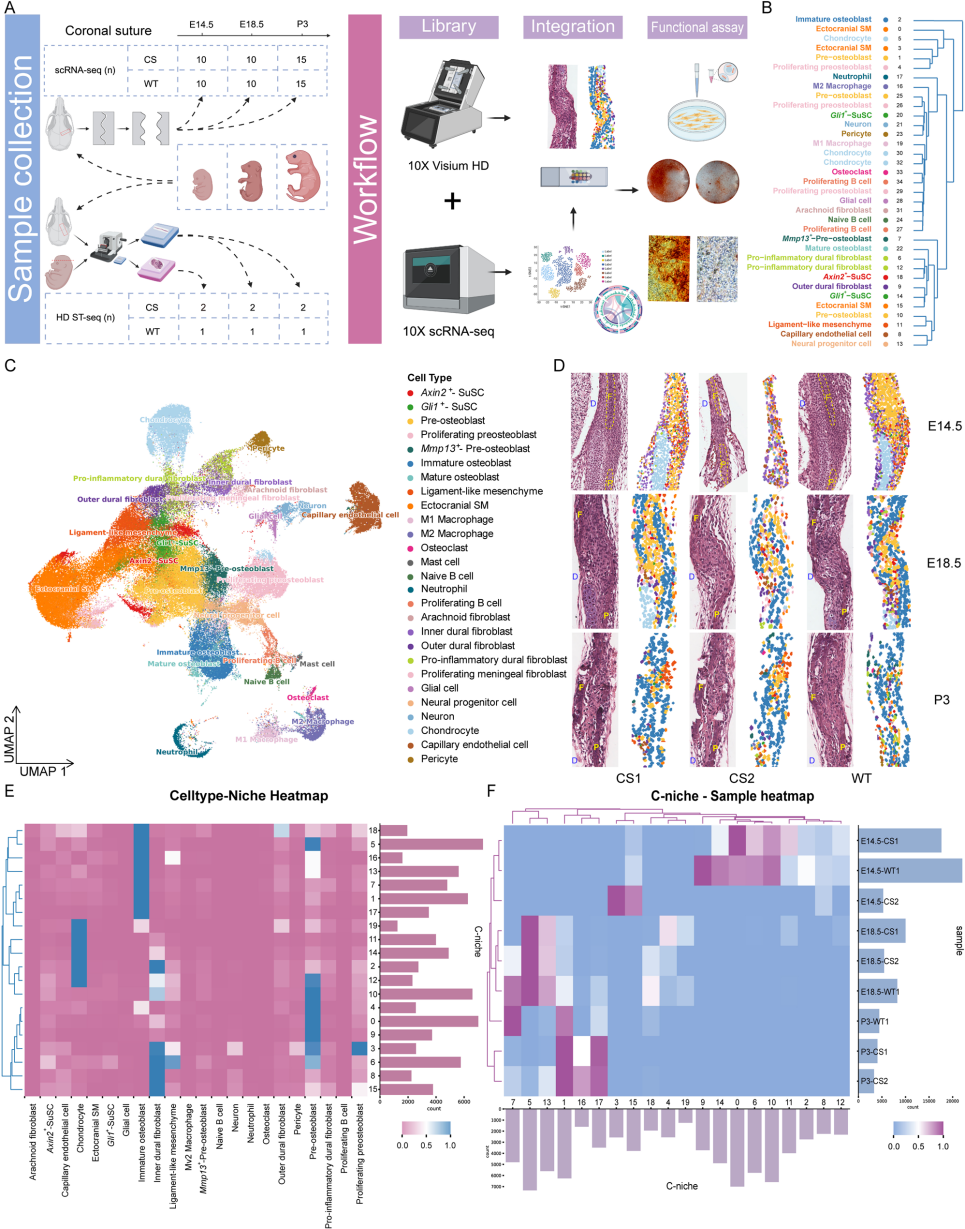
A spatiotemporal map of coronal suture development in craniosynostosis. **A** Workflow schematic of the study. Created in BioRender. Chen, X. (2025) https://BioRender.com/3vcpvth. Functional assay: a focused functional assay on the prioritized candidate *Foxa3*. **B** Dendrogram illustrating scRNA-seq cell clusters’ relatedness. **C** Combined UMAP plot of all scRNA-seq libraries. **D** Spatial mapping of cell types onto hematoxylin and eosin (H&E). Panels share orientation (F: frontal bone; P: parietal bone; D: dural side). For E14.5, F and P labels indicate osteogenic condensations defined by *Sp7* expression. **E** Heatmap of C-niche clusters from HD ST-seq samples, showing the relative proportion of each cell type within each niche. **F** Heatmap of niche composition across HD ST-seq samples, depicting the abundance of each C-niche cluster in each sample

In this study, we collected coronal suture samples from craniosynostosis (CS) and wild-type (WT) mice at three developmental time points: embryonic day 14.5 (E14.5), embryonic day 18.5 (E18.5), and postnatal day 3 (P3). All mice were maintained on a C57BL/6 genetic background with littermate controls employed to minimize technical variability and heterogeneity (Fig. 1A). The CS model mice carry the *Fgfr2*^*C342Y/*+^ mutation, first described by Eswarakumar et al. and widely used as a model of human Crouzon syndrome [12, 13]. These samples were processed and analyzed by single-cell RNA sequencing (scRNA-seq) and Visium High-Definition Spatial Transcriptomics sequencing (HD ST-seq). For scRNA-seq, four libraries were generated at E14.5 and E18.5 (two from CS and two from WT samples), and six libraries were generated at P3 (three from CS and three from WT samples). For HD ST-seq analysis, we generated nine libraries, including two CS samples and one WT sample at each developmental stage. Given the low biological variability among WT mice on the C57BL/6 genetic background, additional replicates were included for the CS group.

For scRNA-seq analysis, cells were retained for further analysis after filtering out mitochondrial and red blood cells (Figure S1A-C). Analyzing all scRNA-seq libraries combined, we identified 35 distinct cell clusters using Louvain clustering in Seurat [14] and subsequently categorized these into 27 cell types based on differentially expressed genes (DEGs) and established markers from previous studies [5, 6, 8, 9, 11, 15–17]. The identified cell populations included SuSCs, chondrocytes, immune cells, osteoclasts, capillary endothelial cells, pericytes, neurons, and glial cells (Fig. 1B, 1C and Figure S1B, Additional file 1).

Visium HD generates subcellular-resolution (2 μm-bin) spatial transcriptomic profiles, enabling capture-region aggregation into single cells [18]. Although morphology-based segmentation and machine-learning annotation have each been applied to subcellular-resolution data, current computational approaches have not fully integrated these capabilities to maximize the biological insights achievable from high-resolution spatial data. We therefore developed SpatialCell (https://github.com/Xinyan-C/SpatialCell), an open-source, modular end-to-end pipeline that integrates histological segmentation, 2 μm-bin aggregation, and machine-learning classification—enabling ROI-aware, scalable spatial transcriptomics analysis. Bin2cell [19] groups adjacent 2 μm bins into cell-scale units via StarDist-based [20] image segmentation, and TopACT [21]—a support vector machine (SVM) [22] classifier trained on our scRNA-seq reference—assigns cell type labels.

In its original publication, TopACT outperformed both RCTD [23] (mean accuracy, M = 0.668 ± 0.010) and a standard fixed-window approach [24] (M = 0.693 ± 0.009), achieving a mean accuracy of M = 0.808 ± 0.006. We incorporated TopACT into our SpatialCell pipeline and applied it to CS and WT samples across three developmental stages, generating cell-level annotations across the tissue. The resulting cell type maps are highly consistent with published in situ hybridization patterns [11, 15, 25]. Notably, for E14.5 samples where morphological ossification is incipient, the spatial accuracy of frontal and parietal bone regions was further confirmed by the expression of the osteogenic marker *Sp7* (Figure S5A), supporting the reliability of our spatial cell classification and single-cell annotation (Fig. 1D, Figures S1C–F, S5B).

**Fig. 2:**
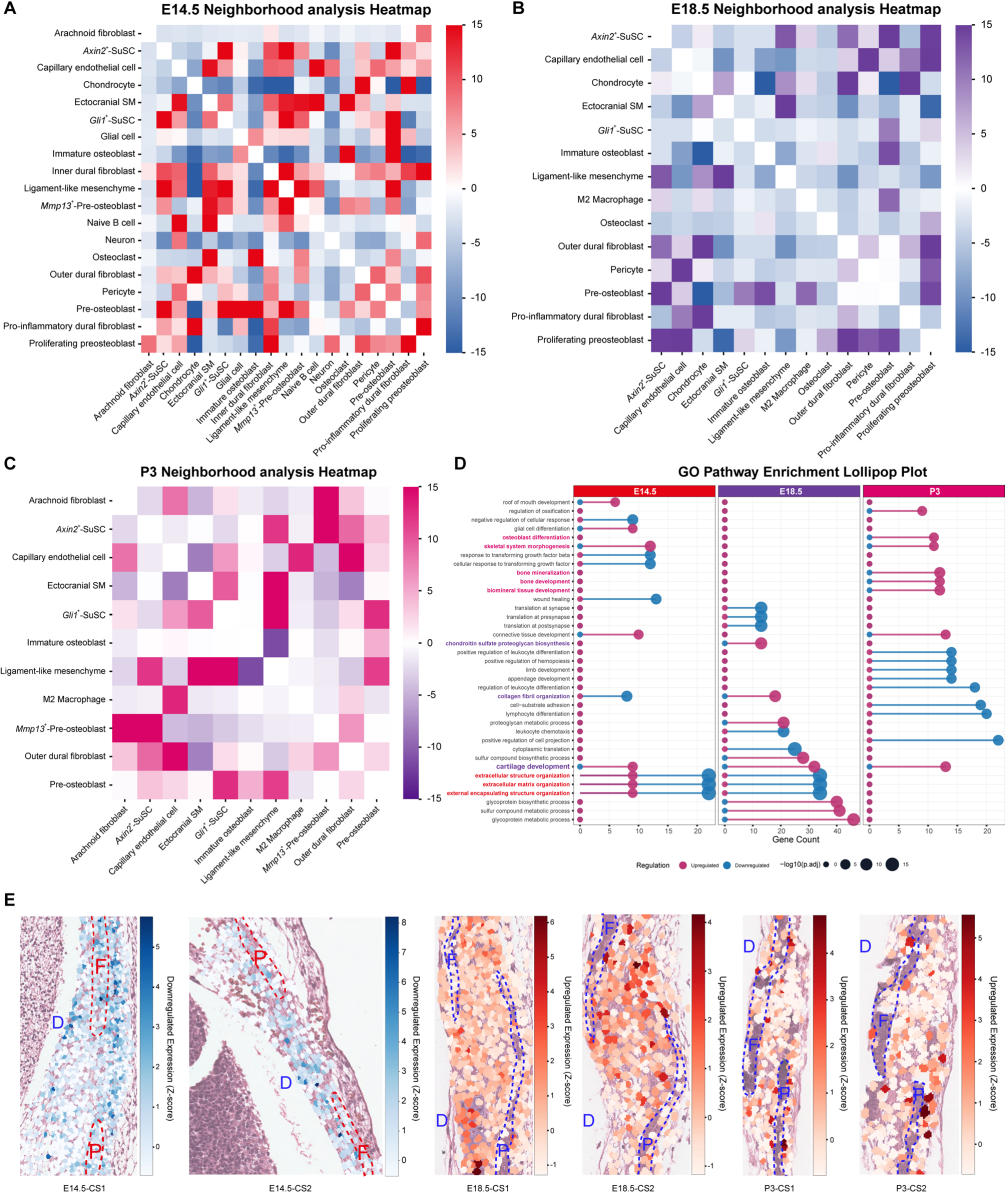
A spatiotemporal map of coronal suture development in craniosynostosis: Neighborhood and Gene Expression Analysis. **A**–**C** Neighborhood-analysis heatmaps of E14.5 (**A**), E18.5 (**B**), and P3 (**C**) HD ST-seq samples, showing z-score–scaled enrichment of cellular neighborhoods. **D** GO-BP enrichment of scRNA-seq DEGs between CS and WT at three developmental stages, performed separately for upregulated and downregulated genes. **E** Spatial metagene mapping at three developmental stages, showing regional enrichment of metagene modules derived from DEGs. F: frontal bone; P: parietal bone; D: dural side

To investigate cellular spatial organization during CS development, we performed niche composition analysis using SOAPy [26] on both CS and WT. K-means clustering of neighborhood composition profiles identified 20 cell niches (C-niches), revealing genotype-specific alterations in spatial architecture across developmental stages. Compared to WT controls, CS samples exhibited stage-specific differences in niche composition. In particular, niches 0, 3, and 9 at E14.5, niches 4, 7, and 18 at E18.5, and niches 16 and 17 at P3 exhibited genotype-specific differences in cell-type abundance between CS and WT, characterized by enrichment of pre-osteoblasts, proliferating pre-osteoblasts, and osteoblast subsets (Fig. 1E, 1F). To characterize the spatial relationships among individual cell types that contribute to these niche differences and to provide a foundation for subsequent cell–cell communication analysis, we performed pairwise proximity analysis across developmental stages (Fig. 2A–C). This analysis revealed that *Gli1*^*+*^- and *Axin2*^*+*^-SuSCs in CS samples progressively shifted their spatial associations from ectocranial and dural fibroblast populations toward pre-osteoblasts and osteogenic mesenchyme across developmental stages. This dynamic reorganization was distinct from WT controls and suggests the early establishment and postnatal expansion of aberrant osteogenic niches in CS.

To elucidate the progression of CS pathogenesis across different developmental stages, we performed Gene Ontology (GO) enrichment analysis on differentially expressed genes (DEGs) between CS and WT mice at each time point. This analysis revealed temporally distinct pathological signatures underlying CS progression. At E14.5, downregulated DEGs in CS mice were primarily associated with disruptions in extracellular matrix (ECM) homeostasis. By E18.5, upregulated DEGs were enriched in pathways associated with chondrogenic activity and ECM organization. At P3, DEGs showed significant enrichment in bone mineralization and osteoblast differentiation pathways (Fig. 2D, Additional file 2). To identify the cellular sources contributing to these pathological processes, we applied BANKSY [27] to analyze metagene expression patterns at single-cell resolution. This method integrates functionally related gene sets into metagene modules to map the spatial distribution of biological processes with cellular precision. At E14.5, DEG-associated metagenes were predominantly expressed in the dura mater and ectocranial suture mesenchyme. At E18.5, elevated chondrogenic activity was detected not only in cartilage regions but also notably within the osteogenic front, suggesting a shift toward endochondral programs [28]. By P3, bone deposition signatures extended into mesenchymal cell regions beyond the osteogenic front, indicating a spatial expansion of ossification activity (Fig. 2E).

### Disrupted SuSC differentiation underlies premature osteogenesis in craniosynostosis

**Fig. 3:**
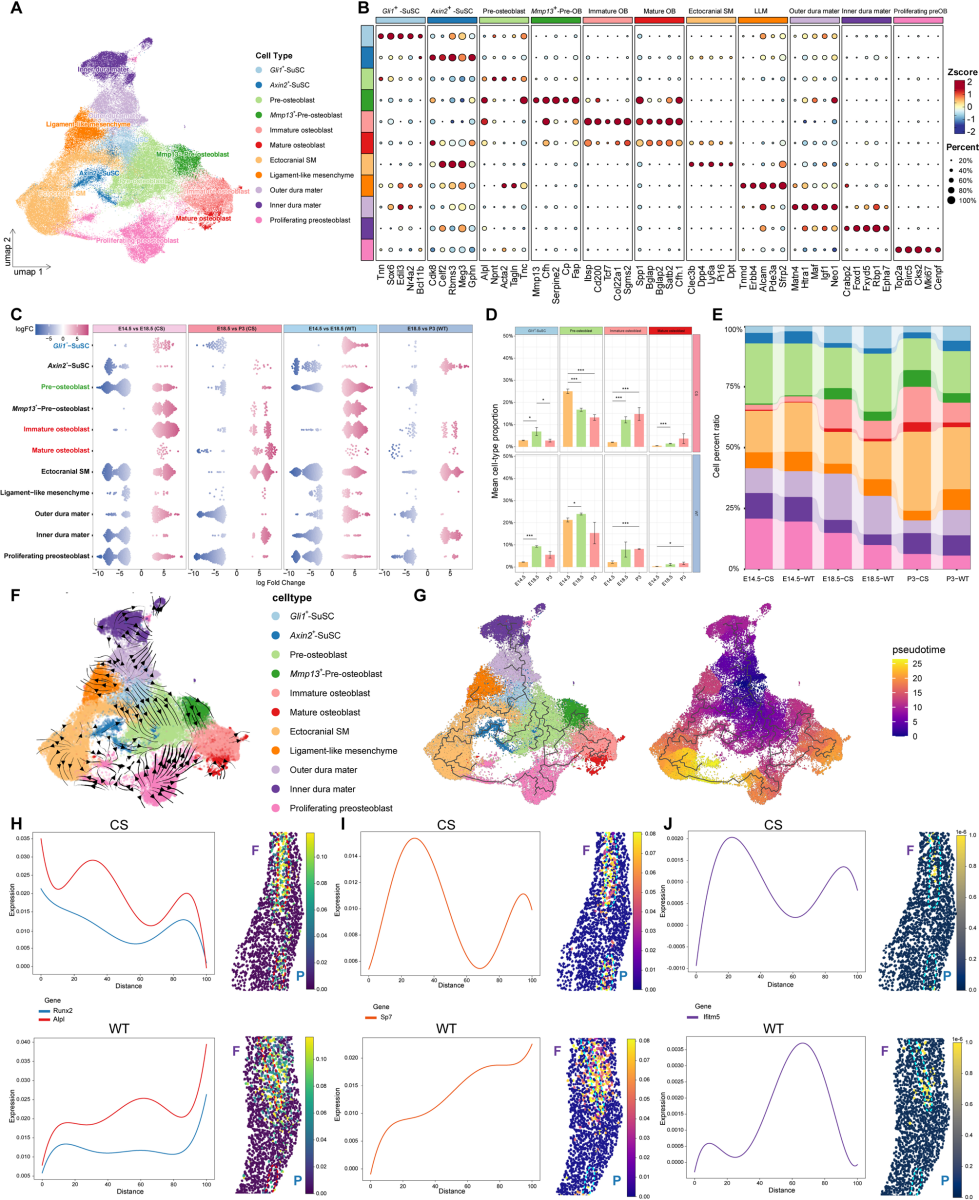
Disrupted SuSC differentiation underlies premature osteogenesis in craniosynostosis. **A**, **B** scRNA-seq suture mesenchymal cell (SM) subclusters: UMAP visualization (**A**) and marker gene dot plot (**B**) (OB, osteoblast; LLM, Ligament-like mesenchyme). C-E. scRNA-seq differential SM abundance between CS and WT across timepoints: **C** Milo analysis beeswarm plot showing neighborhood abundance distribution; blue/red dots indicate significantly decreased/increased abundance, color intensity shows significance degree. **D** Temporal changes in cell-type proportions with pairwise timepoint comparisons (Wilcoxon test, FDR-adjusted): *q < 0.05; **q < 0.01; ***q < 0.001. **E** Sankey diagram of temporal cell-type shifts. **F**, **G** scRNA-seq differentiation trajectories along the SuSC-to-osteoblast axis in CS samples inferred by CytoTRACE (**F**) and Monocle3 pseudotime analysis (**G**), showing progression from SuSC through pre-osteoblast and immature osteoblast to mature osteoblast. **H**–**J** Spatial tendency analysis for osteogenic regulators relative to *Gli1*^+^ -SuSC at E14.5. Each panel shows: left, polynomial regression of gene expression versus distance from SuSC boundary; right, spatial map highlighting regional enrichment. **H** *Runx2*, *Alpl* regression curves; *Alpl* spatial map (SuSC-proximal); **I** *Sp7* (pre-osteoblast); **J** *Ifitm5* (osteoblastic). Panels share orientation (F: frontal bone; P: parietal bone)

Recognizing that disrupted self-renewal and differentiation balance of SuSCs is a hallmark of craniosynostosis [5–8], our study aimed to better understand this dysregulation in Crouzon syndrome. Using scRNA-seq, we performed reclustering on suture mesenchymal cells based on the previous studies [15, 29], specifically targeting Col1a1⁺ cells that concurrently expressed osteogenic markers (*Sp7*⁺ or *Runx2*⁺ or *Alpl*⁺) (Figure S2A). Guided by established markers [11], we classified osteogenic and mesenchymal clusters into 11 subpopulations representing 9 major cell types (Fig. 3A, 3B, Additional file 3): Proliferating preosteoblast(*Top2a*⁺); Pre-osteoblast(*Alpl*⁺ and *Tnc*⁺); Immature osteoblast (*Ibsp*⁺, *Cd200*⁺ and *Tcf7*⁺); Mature osteoblast (*Spp1*⁺); Ectocranial suture mesenchyme (*Clec3b*⁺, *Ly6a*⁺, *Pi16*⁺ and *Dpt*⁺); Ligament-like mesenchyme (*Tnmd*⁺); Dura mater including Outer dura mater (*Matn4*⁺ and *Fxyd5*⁺) and Inner dura mater(*Crabp2*⁺and *Fxyd5*⁺); and SuSCs, defined by the expression of canonical mesenchymal stem cell markers (*Nt5e*⁺, *Thy1*⁺, and *Eng*⁺) and the absence of lineage-specific differentiation markers. SuSCs were further subclassified into *Gli1*⁺ and *Axin2*⁺ subsets (Figure S2B, Additional file 3).

We employed the MiloR [30] to perform differential abundance analysis of cell type proportions between CS and WT across three developmental stages. The analysis revealed that both immature and mature osteoblasts exhibited increased abundance as early as E14.5-E18.5, indicating premature osteogenic activity in CS. Notably, although both *Gli1*⁺ SuSCs and pre-osteoblasts exhibited a progressive decline during CS progression, their temporal patterns differed: pre-osteoblasts declined from E14.5 to E18.5, while *Gli1*⁺ SuSCs showed a delayed reduction occurring between E18.5 and P3 (Fig. 3C). These alterations were statistically validated using Wilcoxon rank-sum tests with false discovery rate (FDR) correction (Fig. 3D, 3E, Figure S2C), underscoring disrupted stem cell population dynamics in CS.

To further understand the differentiation programs underlying the altered population dynamics, we applied CytoTRACE [31, 32] to infer the differentiation potential along the SuSC-to-osteoblast lineage. CytoTRACE revealed a continuous differentiation trajectory along the SuSC-to-osteoblast axis, progressing through SuSC, pre-osteoblast, immature osteoblast, and mature osteoblast stages (Fig. 3F, Figure S2D). This inferred trajectory was further corroborated by pseudotime analysis using Monocle3 [33]. (Fig. 3G, Figure S2E). Notably, pre-osteoblasts reside downstream of SuSCs in both the osteogenic lineage and the pseudotime trajectory, yet they were unexpectedly depleted earlier than SuSCs in CS samples. To explore this paradox, we employed spatial tendency analysis [26]—a regression-based approach that quantifies gene expression patterns relative to anatomical landmarks—using the cranial suture midline, where *Gli1*⁺ SuSCs are located, as the spatial reference axis. We examined the spatial expression of key osteogenic regulators, including *Runx2* and *Alpl*. At E14.5, CS samples showed enrichment of *Runx2* and *Alpl* in *Gli1*⁺ SuSCs, while regions enriched for pre-osteoblasts showed elevated expression of *Sp7* and *Ifitm5*, indicating altered osteoblastic differentiation patterns. In contrast, WT samples displayed spatially ordered differentiation: *Gli1*⁺ SuSCs exhibited low expression of early osteogenic regulators, and osteogenic genes were predominantly expressed in regions distal to SuSCs, corresponding to areas proximal to differentiated osteoblast populations (Fig. 3H, 3I, 3J). These findings suggest that in CS, both *Gli1*⁺ SuSCs and Pre-osteoblasts prematurely acquire downstream osteogenic signatures, driving accelerated osteoblast differentiation.

### Identification of regulatory genes and networks in craniosynostosis

**Fig. 4:**
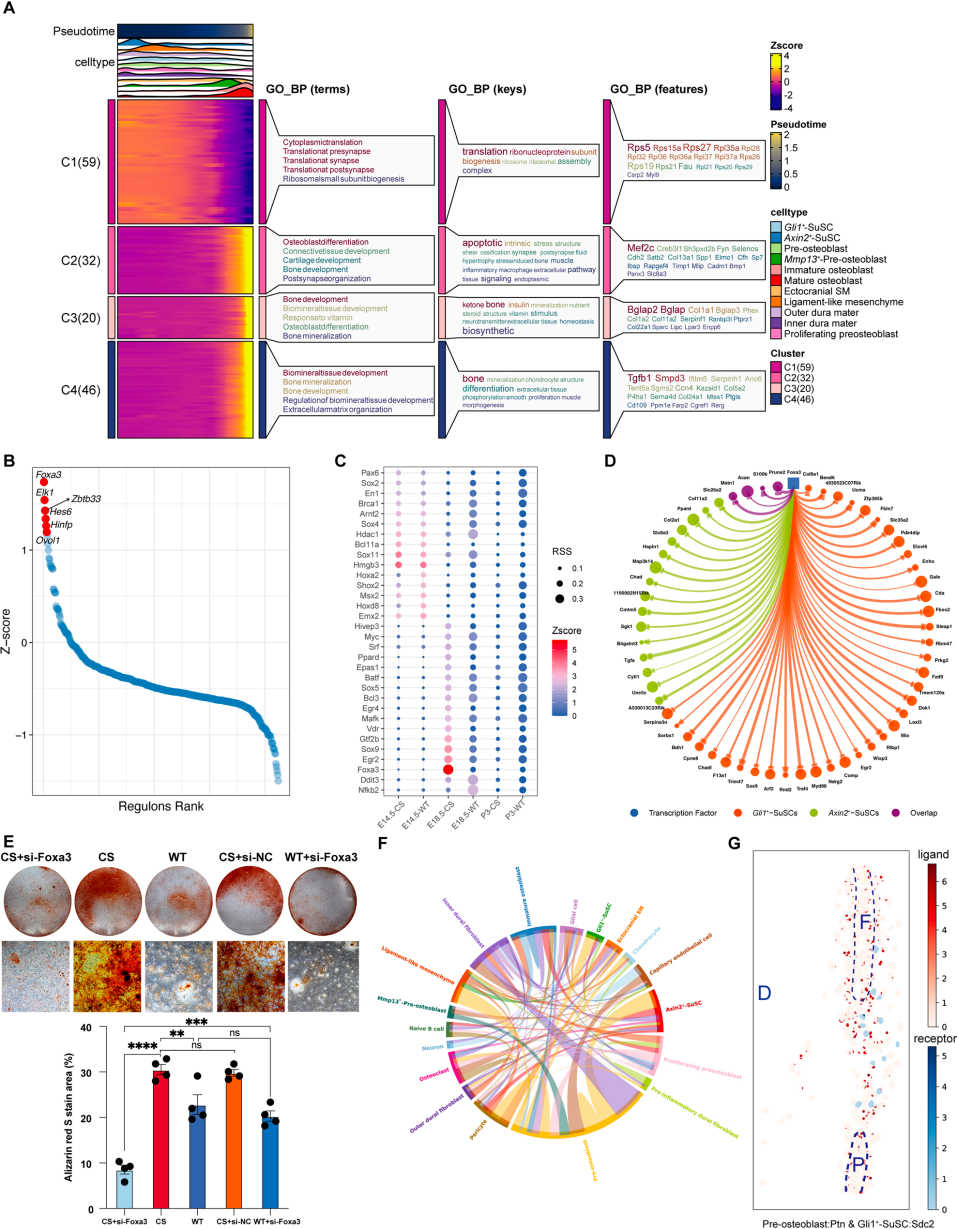
Identification of regulatory genes and networks in craniosynostosis. **A** Heatmap of CS dynamic gene expression along SuSC-to-osteoblast pseudotime trajectory. Four temporal clusters with associated GO biological process terms are shown. **B** Scatter plots of top 6 TF regulons based on Z scores in *Gli1*^+^-SuSCs. **C** Heatmap of regulon specificity scores (RSS) across developmental timepoints and disease conditions in *Gli1*^+^-SuSCs. **D** Transcriptional regulatory network of *Foxa3* in *Gli1*^+^-SuSCs showing target gene interactions. **E** Alizarin Red S staining and quantification of calcium deposition in E18.5 coronal suture mesenchyme cultures from CS and WT mice with *Foxa3* silencing on day 14. Each biological replicate consisted of pooled suture mesenchymal cells from three E18.5 littermates per genotype. Data represent four independent biological replicates (n = 4 pools per group); values are mean ± SEM. Significance determined by one-way ANOVA, **p < 0.01, ***p < 0.001 and ****p < 0.0001. **F**, **G** Spatial cell communication analysis in E14.5 CS. **F** Secretory communication network. Chord diagram showing significant ligand–receptor interactions (strength > 4, p < 0.05) from Visium HD data (SOAPy-ST). Colored sectors represent cell types; chords connect cell-type pairs; chord width indicates the number of significant ligand–receptor pairs. Top 20 most communicative cell types are shown. **G** Ptn-Sdc2 ligand-receptor spatial distribution showing secretory communication. Color intensity indicates normalized expression level. F: frontal bone; P: parietal bone; D: dural side

To investigate transcriptional dynamics along the SuSC-to-osteoblast trajectory, we performed unsupervised clustering of pseudotime-associated genes and identified four temporally distinct expression modules (Fig. 4A). Cluster 1, enriched at early stages, was associated with protein synthesis and growth pathways (e.g., cytoplasmic translation, ribosome biogenesis), marked by ribosomal genes (*Rps5*, *Rps27*, *Rpl35a*) and cytoskeletal regulators (*Csrp2*, *Myl9*). Clusters 2–4 reflected progressive osteogenic maturation: Cluster 2 captured lineage initiation (e.g., *Sp7*, *Ibsp*, *Spp1*; *Mef2c*, *Creb3l1*); Cluster 3 marked active matrix production (e.g., *Bglap*, *Col1a1*, *Col11a2*); and Cluster 4 represented terminal biomineralization, enriched for *Tgfb1*, *Ifitm5*, *Col24a1*. Notably, CS samples exhibited premature co-expression of proliferative and late-stage osteogenic signatures, indicating disrupted temporal regulation of differentiation. In contrast, WT samples showed a simpler, sequential transcriptional pattern dominated by early angiogenesis and late regulation of bone mineralization (Figure S3A).

To further delineate the regulatory mechanisms contributing to the altered differentiation dynamics in CS, we employed SCENIC analysis [34]. This approach nominated *Foxa3* as a candidate transcriptional regulator in both *Gli1*⁺ and *Axin2*⁺ SuSC populations (Fig. 4B, Figure S3B). *Foxa3*, a forkhead pioneer factor with roles in early developmental patterning and metabolism [35], to our knowledge has not been directly examined in craniosynostosis. *Foxa3* regulon activity was elevated at E18.5 in both CS SuSC populations (Fig. 4C, Figure S3C), which may represent a window of heightened susceptibility. GO enrichment of *Foxa3* target genes highlighted processes related to ossification and endochondral bone development (Fig. 4D, Figure S3D), implicating *Foxa3*-linked programs in osteogenic differentiation. To test whether *Foxa3* contributes to the hyper-osteogenic output characteristic of craniosynostosis, we performed ex vivo siRNA knockdown in suture mesenchyme from E18.5 *Fgfr2*^*C342Y/*+^ mice. *Foxa3* knockdown significantly reduced mineralization, as quantified by Alizarin Red S staining, in CS + si-*Foxa3* compared with both CS and CS + si-NC, whereas the reduction in WT was mild and non-significant (Fig. 4E). Consistent with the reduced mineralization, RT-qPCR analyses in the same cultures showed decreased expression of osteogenic markers (*Alpl* and *Sp7*) in the *Foxa3*-silenced group, and ALP staining provided an orthogonal differentiation readout consistent with reduced osteogenic differentiation (Figure S7B-S7D). These results implicate *Foxa3* as a context-dependent contributor to the hyper-mineralization observed ex vivo in the CS background; its in-vivo necessity and sufficiency remain to be determined.

Next, we asked whether the intercellular communication networks also contribute to the dysregulated differentiation observed in CS. We performed Spatial Communication Analysis [26] across three developmental stages (Fig. 4F, 4G, Figure S3E-S3G, Additional file 4). This analysis highlighted multiple signaling pathways including Wnt signaling and other developmental signaling axes. Among these, *Ptn*-*Sdc* interactions in the CS group—specifically involving *Gli1*⁺ SuSCs—were of particular interest, given *Ptn*’s reported role in bone formation [36] and facilitating endochondral ossification by upregulating *Sox9* [37, 38], a key transcription factor that was also enriched in our SCENIC analysis.

In summary, our study proposes a multi-stage regulatory model underlying premature cranial suture fusion in craniosynostosis. At an early developmental stage (E14.5), *Gli1*⁺ SuSCs exhibited aberrant spatial expression of osteogenic markers, including *Runx2* and *Alpl*, indicating an initial disruption of the osteogenic differentiation program. This disruption, coupled with impaired spatial and temporal patterning of differentiation, contributes to the premature fusion of cranial sutures. At a later stage (E18.5), elevated activity of the *Foxa3* regulon coincided with aberrant intercellular signaling events, which further enhanced osteogenic differentiation of SuSCs. Collectively, our findings indicate that craniosynostosis is associated with developmental program dysregulation at multiple stages—spatial disturbances observed at E14.5, *Foxa3*-mediated transcriptional changes at E18.5, and altered intercellular signaling pathways—which may contribute to accelerated osteogenic differentiation within the SuSC niche.

### Suture meningeal fibroblasts contribute to aberrant osteogenesis in craniosynostosis

**Fig. 5:**
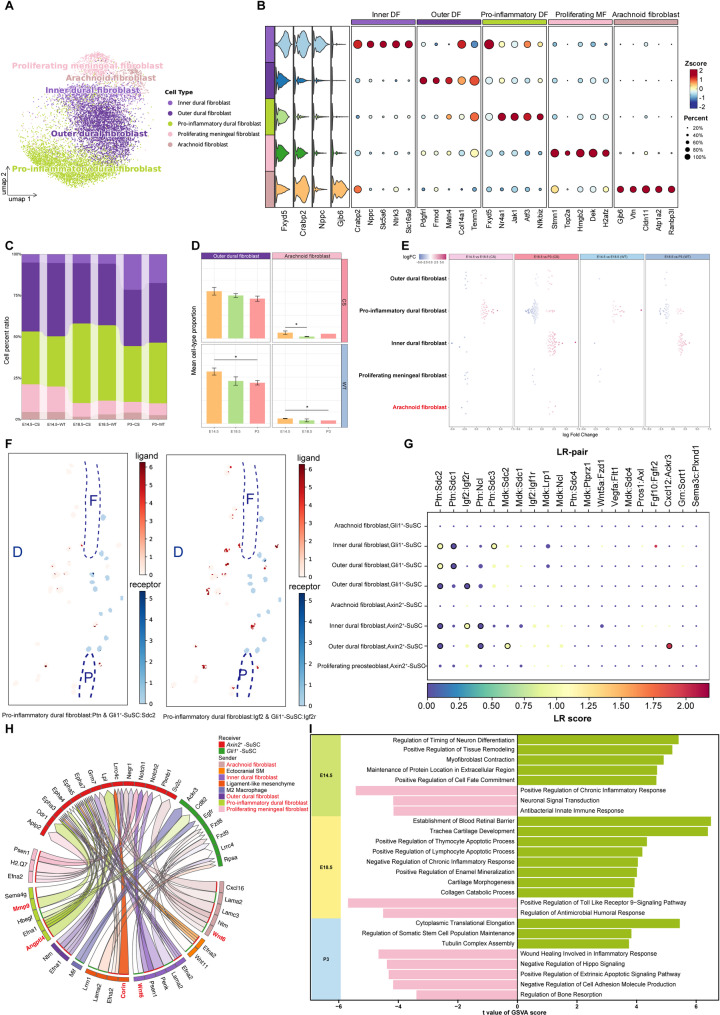
Suture meningeal fibroblasts contribute to aberrant osteogenesis in craniosynostosis. **A**, **B** scRNA-seq Suture meningeal fibroblasts subclusters: UMAP visualization (**A**) and marker gene dot plot (**B**). (DF: dural fibroblast; MF: meningeal fibroblast). **C**–**E** scRNA-seq differential SM abundance between CS and WT across timepoints: **C** Sankey diagram of temporal cell-type shifts. **D** Temporal changes in cell-type proportions with pairwise timepoint comparisons (Wilcoxon test, FDR-adjusted): *q < 0.05. **E** Milo analysis beeswarm plot showing neighborhood abundance distribution; blue/red dots indicate significantly decreased/increased abundance, color intensity shows significance degree. **F**, **G** Spatial cell communication analysis in E14.5 CS: **F** Ligand-receptor spatial distribution showing secretory communication. F: frontal bone; P: parietal bone; D: dural side. **G** Dot plots showing spatial secretory cell–cell communication patterns. **H** Circos plot of top 50 cell–cell interactions between cell types (ligand) and SuSCs (receptor) in E14.5. primary signaling sources are suture meningeal fibroblasts at this stage. **I** Bar plot of GSVA pathway activities in arachnoid fibroblasts across developmental stages comparing CS vs WT samples

Previous studies have suggested that dura mater–derived fibroblasts contribute to cranial suture development and may be involved in the pathogenesis of craniosynostosis. However, their specific role in suture fusion processes and their interactions with other cell populations remain poorly understood [39, 40]. Our scRNA-seq identified five suture meningeal fibroblast subpopulations according to previous studies [11, 15, 41]: inner dural fibroblasts (*Crabp2*⁺, *Nppc*⁺); outer dural fibroblasts (*Matn4*⁺, *Pdgfrl*⁺), Pro-inflammatory dural fibroblasts (*Fxyd5*⁺, *Nr4a1*⁺ and *Jak1*⁺), Proliferating meningeal fibroblasts (*Fxyd5*⁺, *Stmn1*⁺ and *Top2a*⁺) and Arachnoid fibroblasts (*Crabp2*⁺, *Nppc*⁺ and *Gjb6*⁺) (Fig. 5A, 5B).

Pairwise temporal comparisons of cell proportion dynamics revealed similar developmental patterns between CS and WT across most meningeal fibroblast populations. Inner dural fibroblasts showed progressive expansion, pro-inflammatory dural fibroblasts exhibited biphasic dynamics with a peak at E18.5, and both proliferating meningeal and outer dural fibroblasts demonstrated gradual decline (Fig. 5C, 5D). The primary genotype-specific difference was observed in arachnoid fibroblast proportions, as revealed by MiloR analysis: in CS, the abundance of these cells increased from E18.5 to P3 (Fig. 5E).

Spatial Communication Analysis [26] was performed to investigate whether meningeal fibroblasts contribute to SuSC osteogenic differentiation. Only ligand–receptor interactions with statistically significant enrichment (*p* < 0.05) were considered (Fig. 5F, 5G, Figure S4A). Several ligands detected at these stages have been previously reported to promote osteogenic differentiation, including *Igf2* [42] and *Ptn* [37] at E14.5, and *Wnt10b* [43] along with continued *Ptn* expression at E18.5. Given the inherent limitations of spatial transcriptomics—particularly its restricted tissue coverage and reduced sensitivity for detecting low-abundance ligand–receptor interactions—we further employed MultiNicheNet [44] to enhance inference of intercellular communication. This analysis focused on the top 50 ligands that showed significant upregulation in CS (compared to WT) across developmental stages and were predicted to target SuSCs. Among these, *Mmp9* [45, 46]*, Wnt6* [47], *Corin* [48] and *Angptl4* [49] were identified as potential regulators, each previously implicated in osteogenic differentiation (Fig. 5H).

Given the observed shifts in arachnoid fibroblast abundance and the osteoinductive potential of their predicted ligands, we further analyzed their functional alterations across developmental stages using gene set variation analysis (GSVA). At E14.5, CS samples showed upregulation of pathways associated with tissue remodeling and myofibroblast contractile pathways, accompanied by enhanced extracellular matrix organization and altered programs of cell fate specification. Concurrently, chronic inflammatory responses and antimicrobial defenses were suppressed, suggesting compromised immune surveillance despite preserved tissue remodeling capacity and support for neurodevelopmental processes. At E18.5, arachnoid fibroblasts in CS exhibited increased activity in cartilage development and collagen catabolism, along with enrichment of pathways related to lymphocyte apoptosis. In parallel, downregulation of TLR9 signaling and humoral antimicrobial responses suggested a coordinated shift toward structural remodeling and immune modulation. By P3, pathways related to bone resorption, inflammatory wound healing, and cell adhesion were downregulated, indicating progressive impairment in tissue remodeling and skeletal homeostasis (Fig. 5I, Additional file 5).

### Altered immune responses contribute to aberrant osteogenesis in craniosynostosis

**Fig. 6:**
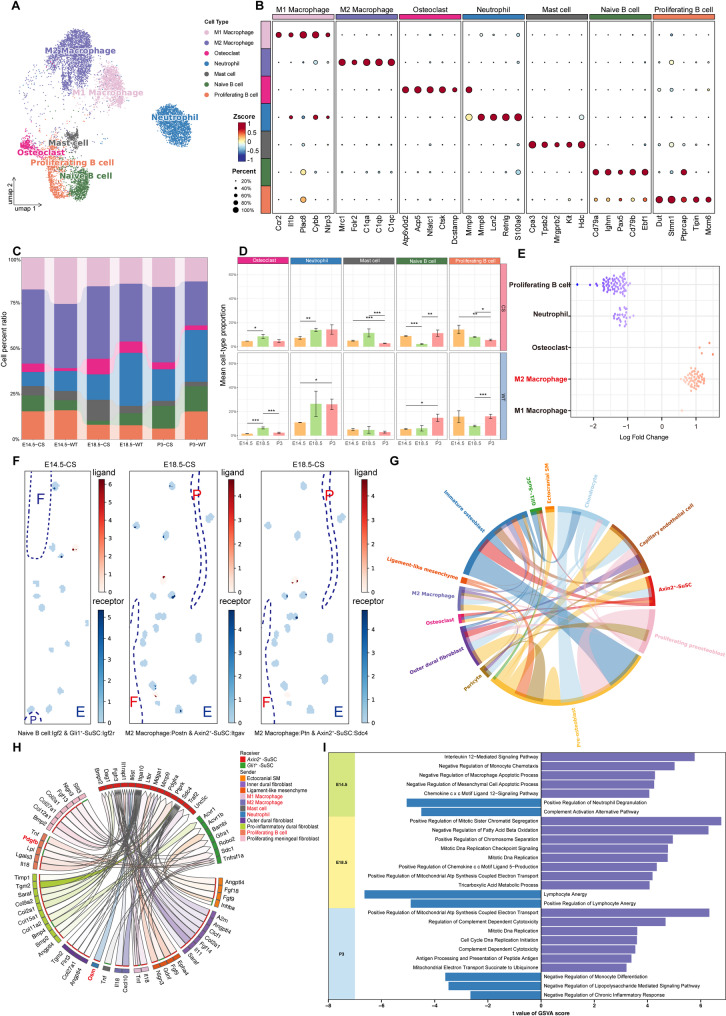
Altered immune responses contribute to aberrant osteogenesis in craniosynostosis. **A**, **B** scRNA-seq Suture immune cells subclusters: UMAP visualization (**A**) and marker gene dot plot (**B**). **C**–**E** scRNA-seq differential cell abundance between CS and WT across timepoints: **C** Sankey diagram of temporal cell-type shifts. **D** Temporal changes in cell-type proportions with pairwise timepoint comparisons (Wilcoxon test, FDR-adjusted): *q < 0.05; **q < 0.01; ***q < 0.001. **E** Milo analysis beeswarm plot showing neighborhood abundance distribution of P3 CS vs WT; blue/red dots indicate significantly decreased/increased abundance, color intensity shows significance degree. **F**, **G** Spatial cell communication analysis in E14.5 and E18.5 CS. **F** Ligand-receptor spatial distribution showing targeted immune-to-SuSC secretory communication. Color intensity indicates normalized expression level. F: frontal bone; P: parietal bone; E: ectocranial side. **G** Secretory communication network of E18.5. Chord diagram showing significant ligand–receptor interactions (strength > 4, p < 0.05) from Visium HD data (SOAPy-ST). Colored sectors represent cell types; chords connect cell-type pairs; chord width indicates the number of significant ligand–receptor pairs. Top 20 most communicative cell types are shown. **H** Circos plot of top 50 cell–cell interactions between cell types (ligand) and SuSCs (receptor) in E18.5. Compared with Circos plot of E14.5 in Fig. 5H, primary signaling sources shift from suture meningeal fibroblasts to immune cells. **I** Bar plot of GSVA pathway activities in M2 macrophage across developmental stages comparing CS vs WT samples

Recent studies have uncovered skull–meninges channels and brain-border immune niches that enable immune cell trafficking between the calvarial bone, meninges, and brain, challenging the classical concept of central nervous system (CNS) immune privilege [50–52]. While these findings highlight the immunological activity at the skull–CNS interface, the involvement of immune cells in craniosynostosis has been suggested but remains largely uncharacterized [53, 54]. Through scRNA-seq analysis, we identified seven distinct immune cell populations within the coronal suture: M1 macrophages [55] (*Ccr2*⁺, *Il1b*⁺, *Plac8*⁺, *Cybb*⁺, *Nlrp3*⁺), M2 macrophages [56] (*Mrc1*⁺, *Folr2*⁺, *C1qa*⁺, *C1qb*⁺, *C1qc*⁺), osteoclasts (*Atp6v0d2*⁺, *Acp5*⁺, *Nfatc1*⁺, *Ctsk*⁺, *Dcstamp*⁺), neutrophils (*Mmp9*⁺, *Mmp8*⁺, *Lcn2*⁺, *Retnlg*⁺, *S100a9*⁺), mast cells (*Cpa3*⁺, *Tpsb2*⁺, *Mrgprb2*⁺, *Kit*⁺, *Hdc*⁺), naive B cells (*Cd79a*⁺, *Ighm*⁺, *Pax5*⁺, *Cd79b*⁺, *Ebf1*⁺), and proliferating B cells (*Dut*⁺, *Stmn1*⁺, *Ptprcap*⁺, *Tipin*⁺, *Mcm6*⁺) (Fig. 6A, 6B, Figure S4B, S4C), underscoring their diversity in the suture niche.

Pairwise temporal comparisons across the three developmental stages revealed distinct immune cell population dynamics in CS samples. Specifically, CS samples exhibited significant changes in mast cell proportions between E18.5 and P3, U-shaped temporal changes in naive B cell proportions (with significant differences between E14.5 and E18.5, and between E18.5 and P3), and significant decreases in proliferating B cell proportions between E14.5 and P3. Additional temporal variations were observed in neutrophil and osteoclast populations, with specific significant differences at individual timepoints (Fig. 6C, 6D). Cross-genotype analysis using MiloR and Wilcoxon testing with FDR correction further revealed a significant increase in M2 macrophage proportions in CS at P3 (Fig. 6E, Figure S4D, S4E).

To assess whether immune-derived signals may influence SuSC osteogenic commitment in CS, we performed spatial communication analysis and prioritized secretory ligand–receptor pairs with immune cells as senders and *Gli1*⁺ /*Axin2*⁺ SuSCs as receivers. This analysis identified candidate osteogenesis-related interactions (p < 0.05), including naive B cell–derived IGF2 signaling [42] to *Gli1*⁺ SuSCs at E14.5 and M2 macrophage–derived *Postn*–*Itgav* and *Ptn*-*Sdc4* signaling to *Axin2*⁺ SuSCs at E18.5 (Fig. 6F). In parallel, we summarized the dominant secretory communication links at E18.5 at the cell-type level using a chord diagram, providing an unbiased overview that is not restricted to immune-SuSC interactions (Fig. 6G). To obtain a more comprehensive view of immune cell-mediated communication, we applied MultiNicheNet analysis to identify stage-specific ligands potentially involved in regulating SuSC differentiation. Upon comparing signals influencing SuSCs across developmental stages, we noted a shift from suture meningeal fibroblasts as primary signaling sources at E14.5 to immune cells playing a pivotal role at E18.5. Several immune-derived factors with previously reported roles in promoting osteogenic differentiation were identified, including *Mif* [57] at E14.5; *Pdgfb* [58], *Osm* [59] at E18.5; and *Efnb2* [60] and *Pdgfb* at P3 (Fig. 6H, Figure S4F, S4G).

Given both the increased M2 macrophage abundance and their recurrent involvement in osteogenic signaling to SuSCs, we subsequently performed GSVA to evaluate the temporal dynamics of their functional states. At E14.5, CS samples exhibited activation of IL-12 and CXCL12 signaling pathways, with concurrent suppression of apoptotic processes in both macrophages and mesenchymal cells, as well as reduced monocyte chemotaxis. By E18.5, functional profiles shifted to show upregulation of the TCA cycle and mitochondrial ATP synthesis, while fatty acid β-oxidation was inhibited. Pathways associated with cell proliferation, including mitotic DNA replication and chromosome segregation, were upregulated, with concurrent elevation of Ccl5 production and reduction in lymphocyte anergy. At P3, CS samples demonstrated aberrant immune activation, characterized by enhanced complement-dependent cytotoxicity and upregulation of antigen processing and presentation pathways, while monocyte differentiation was simultaneously downregulated. Concomitantly, regulatory mechanisms of inflammation appeared disrupted, as evidenced by reduced LPS tolerance and impaired suppression of chronic inflammation (Fig. 6I, Additional file 6). These sequential alterations in M2 macrophage function—progressing from selective immune modulation through metabolic reprogramming and proliferative activation to ultimately dysregulated immune responses—suggest a distinct temporal pattern potentially contributing to CS pathogenesis.

## Discussion

In this work, we present a cross-genotype, multi–timepoint spatiotemporal atlas of SuSCs in the *Fgfr2*^*C342Y/*+^ mouse model and wild-type controls, integrating single-cell RNA-seq with 2 μm-resolution Visium HD spatial transcriptomics. This integrative framework, supported by our open-source SpatialCell pipeline for morphology-driven segmentation and machine learning-based annotation, allowed us to resolve cellular heterogeneity at near–single-cell resolution. Leveraging this resource, we provide a spatiotemporal single-cell and spatial atlas of the developing coronal suture in the *Fgfr2*^*C342Y/*+^ craniosynostosis model. Our analyses highlight stage-dependent alterations in SuSC states and their surrounding niches, and generate testable hypotheses about regulatory programs and intercellular signaling that may precede suture fusion.

Consistent with prior studies indicating that craniosynostosis can involve early defects in suture patterning and lineage specification, rather than being explained solely by increased osteoblast production [62–64], our atlas provides a stage- and niche-resolved view of how the disease state disrupts the timing and coordination of developmental programs across the SuSC-to-osteogenic continuum. The paradoxical depletion of pre-osteoblasts before their upstream SuSCs, combined with the aberrant co-expression of proliferative and terminal differentiation signatures, indicates that craniosynostosis fundamentally disrupts the temporal coordination of developmental programs rather than merely accelerating normal processes. Our working model is that elevated *Foxa3* activity at E18.5 may bias SuSCs toward osteogenic programs, which could contribute to premature ossification, spatial disorganization of differentiation gradients, and multi-level signaling changes. This hypothesis is generated by integrative analyses and ex vivo perturbation and will require in vivo testing.

While lineage tracing and ablation of *Gli1*⁺ and *Axin2*⁺ cells confirmed their roles in suture maintenance and osteogenic differentiation [5, 6], these genetic approaches lack the spatial and temporal resolution to pinpoint the exact window and micro-niche of SuSC commitment. Our high-resolution atlas pinpoints an early window (E14.5) and niche locale (SuSC midline) at which *Gli1*⁺ cells first exhibit upregulation of osteogenic markers (e.g., *Runx2*, *Alpl*), and a later window (E18.5) during which *Foxa3* regulon activity peaks. This early midline activation suggests the deviation occurs at the level of spatial patterning—i.e., osteogenic programs encroach into the SuSC niche that normally maintains an undifferentiated reservoir—thereby weakening the spatial constraints that coordinate orderly differentiation gradients. Moreover, earlier work hinted at broad transcriptional shifts in disease but did not identify upstream regulators [61–63]. By contrast, SCENIC analysis implicates *Foxa3*—a factor not previously linked to suture biology—as a driver of this fate misregulation, a function we further validated through targeted perturbation assays in ex vivo calvarial cultures. Finally, whereas prior reports described increased Wnt signaling [64] and enhanced BMP pathway activity [65] in craniosynostosis, our Spatial Communication Analysis and MultiNicheNet uncover a previously underappreciated *Ptn*-*Sdc* signaling axis and identify ligands from immune cells and meningeal fibroblasts that potentially synergize with *Foxa3* to drive osteoblast differentiation. Together, these findings extend earlier observations by defining when, where, and how SuSCs are hijacked in disease.

Beyond intrinsic SuSC transcriptional dysregulation, we explored how distinct microenvironmental compartments—meningeal fibroblasts and immune cells—coordinate to drive aberrant developmental reprogramming in craniosynostosis. Prior work characterized the transcriptional heterogeneity and temporal dynamics of suture meningeal fibroblasts [15, 40, 41], but lacked spatial insight into how these cells signal to SuSCs. Leveraging our spatiotemporal atlas, Spatial Communication Analysis and MultiNicheNet further highlight arachnoid-derived ligands (*Mmp9*, *Wnt6*, *Corin*, *Angptl4*) as candidate paracrine cues instructing SuSC-to-osteoblast differentiation. Extending our atlas to the immune compartment—a domain seldom explored mechanistically in craniosynostosis [52–54, 66]—we applied the same spatial frameworks to uncover spatially and temporally resolved signaling interactions between immune cells and SuSCs. At E14.5, *Mif* secretion by naive B cells emerges as a putative paracrine regulator of early SuSC activation; by E18.5, *Postn*–*Itgav*/*Itgb5* crosstalk between M2 macrophages and *Axin2*⁺ SuSCs may potentiate osteogenic commitment. GSVA-based functional profiling of macrophages reveals dynamic shifts in cytokine signaling, metabolic pathways, and proliferative cues that coincide with SuSC osteogenic gene expression. Together, these data reveal how suture meningeal fibroblast and immune niches interact with intrinsic transcriptional programs to reprogram SuSC fate.

Building on prior single-cell studies of skeletal tissues, our work extends the spatial and temporal resolution of SuSC analysis into a disease context. While earlier scRNA-seq analyses characterized SuSC heterogeneity in healthy cranial sutures [4, 8, 11, 15], and spot-based spatial methods conflated signals from multiple cell types [9, 67], we combine single-cell transcriptomes with subcellular spatial data across wild-type and craniosynostotic sutures. This dual-modality strategy uncovers genotype-specific niche remodeling invisible to conventional approaches, yielding new insights into disease-associated SuSC dynamics. Moreover, whereas *Gli1*⁺ and *Axin2*⁺ populations were previously defined as SuSC markers [5, 6], our analyses reveal that these subsets follow distinct differentiation trajectories and spatial distributions in craniosynostosis. By situating our findings within this foundational literature, we validate established SuSC identities and reveal their dynamic misregulation in craniosynostosis.

Our craniosynostosis model is driven by an activating FGFR2 mutation; we therefore asked whether SuSC transcriptomes reflect expected changes in canonical FGFR2 downstream signaling. Indeed, in *Gli1*⁺ SuSCs, pathway enrichment analysis identified MAPK and PI3K–Akt signaling as significantly enriched at E18.5 (Figure S6) among CS-associated upregulated genes. These pathway-level shifts are consistent with prior mechanistic studies in syndromic craniosynostosis models indicating that aberrant FGFR2 activity engages the Ras/ERK–MAPK axis during coronal suture development, and that genetic uncoupling of key adaptor signaling or pharmacological inhibition of FGFR/ERK signaling can mitigate premature suture fusion [68–70]. In parallel, we previously showed that hyperactivation of the stress-activated p38α MAPK branch induces a senescence program in cranial suture progenitor cells and contributes to craniosynostosis-associated phenotypes, and that genetic or pharmacological attenuation of p38α/Mapk14 signaling mitigates disease severity [71].

The integration of morphology-driven segmentation with machine-learning classification addresses a persistent challenge in spatial transcriptomics: achieving accurate cell type annotation at subcellular resolution while maintaining computational scalability. Previous approaches have relied on either segmentation-free deconvolution methods or image-based segmentation without transcriptomic validation, each with inherent limitations in cell boundary definition and annotation accuracy. Our unified workflow demonstrates that combining these complementary approaches generates spatial cell-type classifications that align with established in situ expression patterns. This methodological framework may inform future developments in spatial omics, particularly for complex tissue architectures where traditional spot-based approaches cannot resolve cellular heterogeneity.

While this study provides a comprehensive view of SuSC behavior in craniosynostosis, several limitations should be acknowledged. First, although the integration of Visium HD spatial transcriptomics and Bin2cell segmentation enabled near cell-scale resolution, the reconstructed transcriptional units are computationally inferred and may not precisely reflect actual cell boundaries, especially in densely packed regions. Second, although our murine model recapitulates key genetic and developmental features of human craniosynostosis, species-specific anatomical or molecular differences may constrain the translational relevance of our findings. Third, our perturbation experiments provide ex vivo support that *Foxa3* can modulate osteogenic capacity; temporally controlled in vivo perturbations within the suture niche will be required to determine whether *Foxa3* and the predicted ligand–receptor circuits are necessary or sufficient for craniosynostosis-like outcomes. Specifically, inducible and SuSC-targeted genetic perturbations would provide a rigorous route to achieve cell-type specificity and to test these candidate regulators and niche interactions in vivo. Fourth, immune- and meningeal/dural-cell findings are largely computationally inferred and are presented as hypothesis-generating. Tissue-level validation and in vivo functional tests will be needed to confirm these changes and determine their causal relevance.

## Conclusions

In summary, our study provides a spatiotemporal single-cell and high-resolution spatial transcriptomic atlas of coronal suture development in the *Fgfr2*^*C342Y/*+^ mouse model of Crouzon syndrome. The results suggest that craniosynostosis arises from disrupted temporal coordination of SuSC differentiation rather than simple acceleration of osteogenesis. This atlas identifies *Foxa3* and niche-derived cues as candidate regulators and establishes a resource and analytical framework for temporally and spatially targeted studies of cranial suture biology.

## Materials and methods

### Animals

All animal procedures were approved by the Ethics Committee of the Chinese Academy of Medical Sciences and Peking Union Medical College (Approval No. 2023-A-79; 28 August 2023) and performed in accordance with institutional/national guidelines and the Guide for the Care and Use of Laboratory Animals. All mice were maintained on a C57BL/6J background. *Fgfr2*^*C342Y/*+^ mice were generated by CRISPR/Cas9‐mediated introduction of a Cys361Tyr point mutation into the mouse *Fgfr2*-215 transcript (ENSMUST00000122054.8), corresponding to the human Cys342Tyr variant [12, 13], by Collective Pharmachem Biotechnology Co., Ltd. (Nanjing, China). We re-generated the *Fgfr2*^*C342Y/*+^ allele because live animals or embryos carrying the original line were not importable to our facility during the study period due to regulatory and biosafety constraints. CRISPR/Cas9 allowed us to introduce the same missense change on our local barriered colony while maintaining animal welfare and compliance. This CRISPR-based line was previously phenotypically validated by micro-CT, skeletal staining, and histology [13]. To further confirm successful replication of the craniosynostosis phenotype in our current colony, we performed micro-CT imaging of age-matched *Fgfr2*^*C342Y/*+^ and WT mice at P14 and P30, which recapitulated the expected coronal suture fusion in mutants (Figure S7A). To obtain embryos and postnatal offspring at defined developmental stages, *Fgfr2*^*C342Y/*+^ heterozygous males from our colony were sent to the Shanghai Model Organisms Center (Shanghai, China), where in vitro fertilization and embryo transfer were performed using C57BL/6J females (strain no. SM-001). This approach was adopted because *Fgfr2*^*C342Y/*+^ males exhibit reduced fertility, and large-scale timed breeding was required to obtain sufficient embryos and offspring for experimental analyses within a defined developmental window. The resulting offspring were used for colony establishment and subsequent experiments. Animals were housed under a 12-h light/12-h dark cycle at 20–22 °C and 30–70% relative humidity, with ad libitum access to food and water. Genotyping was performed by PCR analysis of genomic DNA extracted from tail biopsies. Embryos were collected at E14.5 and E18.5 without survival procedures; following maternal CO₂ euthanasia (20–30% chamber volume/min), embryos were euthanized by decapitation, and death was confirmed prior to tissue collection. Postnatal pups (P3) were humanely euthanized by CO₂ inhalation (20–30% chamber volume/min) until loss of righting reflex and cessation of respiration and heartbeat, followed by cervical dislocation as a secondary physical method to ensure death. No anaesthesia was administered to animals in this study, as no survival procedures or live-animal procedures requiring anaesthesia were performed. All embryonic and postnatal tissue collections, as well as subsequent imaging procedures, were carried out only after euthanasia. The work has been reported in line with the ARRIVE guidelines 2.0.

### Coronal suture dissociations

Coronal sutures were collected from *Fgfr2*^*C342Y/*+^ and wild-type littermates at E14.5, E18.5 and P3. scRNA-seq libraries were generated from two replicates per genotype at E14.5 and E18.5 and three replicates at P3; spatial transcriptomics was performed with two *Fgfr2*^*C342Y/*+^ and one wild-type replicate at each time point. Skull caps were removed in ice-cold PBS and coronal sutures microdissected under a stereomicroscope. For scRNA-seq, frontal and parietal bones were separated to expose the suture, five sutures per replicate were pooled for a library and both sexes were combined for processing. For HD ST-seq, coronal sutures were fixed in 4% paraformaldehyde in PBS for 12–24 h at room temperature, then dehydrated through graded ethanol (70%, 95%, 100%; 30 min each) and cleared twice in xylene (30 min each). Samples were infiltrated with molten paraffin (58 °C) under vacuum in two 30-min cycles, placed into embedding molds filled with fresh paraffin, and cooled on a chilled plate to solidify. Resulting FFPE blocks were stored at room temperature until sectioning. To ensure precise capture of the coronal suture at E14.5, samples were sectioned transversely parallel to the reference plane connecting the pupil and the midpoint of the outer ear, at a height approximately 1 mm superior to the eye.

### scRNA-seq library preparation

Gel beads containing cell barcodes and unique molecular identifiers (UMIs) were combined with single-cell suspensions at a concentration approaching saturation, ensuring each cell was individually encapsulated within a Gel Bead-in-Emulsion (GEM). Following exposure to cell lysis buffer, polyadenylated mRNA molecules hybridized to bead-attached oligonucleotides. Beads were then recovered into a single tube for reverse transcription. During reverse transcription, each mRNA transcript was converted into cDNA and simultaneously tagged at its 5’ end (corresponding to the original 3’ end of mRNA) with the cell barcode and UMI. Subsequently, the bead-bound cDNA underwent second-strand cDNA synthesis, adaptor ligation, and universal amplification according to the standard manufacturer’s protocol (10 × Genomics, CG000206 Rev D). The sequencing libraries were prepared to specifically enrich for transcript 3’ ends linked with cell barcodes and UMIs. Final library quality was assessed using an Agilent Bioanalyzer 2100 with High Sensitivity DNA Chips and quantified by the Qubit High Sensitivity DNA Assay (Thermo Fisher Scientific). Libraries were sequenced using paired-end sequencing (2 × 150 bp) on an Illumina NovaSeq 6000 platform. Sequencing depth averaged 28,255 ± 6964 reads per cell across all 14 libraries (range: 17,569–41,318 reads per cell).

### Spatial transcriptomics library preparation (FFPE-HD)

Formalin-fixed paraffin-embedded (FFPE) coronal suture sections from *Fgfr2*^*C342Y/*+^ (CS) and wild-type (WT) C57BL/6 mice at E14.5, E18.5, and P3, passing RNA quality control (DV200 > 30%), were processed using the 10 × Genomics Visium HD Spatial Gene Expression platform for FFPE tissue. For each developmental stage, two CS and one WT replicate 5 µm-thick tissue sections were mounted onto a single Visium HD slide (10 × Genomics) in a 2CS + 1WT configuration, baked at 42 °C for 3 h, and dried overnight in a desiccator at room temperature. Slides were deparaffinized by incubation at 60 °C for 2 h, followed by immersion in xylene and rehydration through a graded ethanol series. Hematoxylin and eosin (H&E) staining was performed using Mayer’s hematoxylin (Millipore Sigma), Bluing Reagent (Dako, Agilent), and alcoholic eosin (Millipore Sigma). Slides were scanned under a microscope to document tissue morphology. Subsequently, RNA crosslinks were reversed by incubation in 0.1 N HCl followed by TE buffer (pH 9.0). The slides were then incubated with the Mouse Whole Transcriptome Probe Panel (10 × Genomics). Each probe pair comprised a 5’ probe with a Small RNA Read 2S sequence and a 3’ probe containing a poly-A tail. Probe pairs hybridized to target RNAs were ligated to form single-stranded ligation products. Samples were treated with RNase and permeabilized to release these ligation products. Released ligation products were captured by poly(dT) capture probes precoated on the Visium slide, which included sequences for Illumina Read 1, spatial barcodes, and unique molecular identifiers (UMIs). Capture probes were then extended to generate spatially barcoded ligation products, released from the slide, and subjected to sample indexing PCR, final library preparation, and sequencing. Final Visium Spatial Gene Expression libraries were prepared for Illumina paired-end sequencing, with P5 and P7 adapters flanking the libraries. Libraries were sequenced on an Illumina NovaSeq 6000 platform using paired-end sequencing (Read 1: 16-bp spatial barcode + 12-bp UMI; Read 2S: ligated probe inserts) at a depth of ~ 50,000 reads per spot.

### Single-cell sequencing data processing

Single-cell RNA sequencing data were aligned to a customized mouse reference genome (GRCm38 for E14.5 and E18.5, GRCm39 for P3) using CellRanger. The raw gene expression matrix contained 157,954 cells and 27,468 genes. Data normalization was conducted using SCTransform [72], which simultaneously performs normalization, variance stabilization, and identifies 3000 highly variable genes per sample. Batch effect correction across samples was achieved using Harmony integration [73]. 30 principal components were used for subsequent dimensional reduction and clustering analysis. Cell cycle phase assignment was performed using established S-phase and G2/M-phase gene signatures. Doublet detection was implemented using scDblFinder to identify potential cell doublets based on transcriptomic profiles. Dimensionality reduction for visualization employed both t-distributed stochastic neighbor embedding (t-SNE) and Uniform Manifold Approximation and Projection (UMAP) based on the Harmony-corrected embeddings. Unsupervised clustering was performed using the Louvain algorithm with a resolution of 0.8, resulting in 35 distinct clusters. Differential gene expression analysis was conducted using the Wilcoxon rank-sum test through FindAllMarkers function [14] on the SCT-normalized data after PrepSCTFindMarkers preprocessing. Significantly enriched genes were defined as those with adjusted p-value < 0.05, log fold change > 0.25, and expression in at least 10% of cells within the cluster. Marker genes were ranked by pct_diff (the absolute difference between pct.1 and pct.2) in descending order, followed by avg_log2FC. The cluster identified as erythroid progenitor cells—characterized by high expression of *Car2*, *Slc25a21*, *Ermap*, *AI662270*, and *Mt2*—was excluded. Suture meningeal fibroblasts and immune cells were then re-clustered following the same pipeline used for normalization, variable feature selection, batch correction, dimensionality reduction, and unsupervised clustering, with the clustering resolution set to 0.5. Any cluster failing to exhibit the appropriate lineage markers (immune or suture meningeal fibroblast) was filtered out. In total, 142,579 cells and 27,468 genes across all libraries were retained for downstream analyses.

### Cellular abundance and composition analysis

Cellular abundance analysis was performed using MiloR (v2.2.0) [30] and statistical pipelines. A k-nearest neighbors (KNN) graph was constructed from the Harmony-integrated latent space of the Seurat object using 30 principal components and k = 40. Neighborhoods were defined using the makeNhoods function with a proportion of 0.1. Cell counts per neighborhood were compared between conditions using a generalized linear model (GLM) implemented in MiloR. Differentially abundant neighborhoods with spatial FDR < 0.1 were considered significant. Cell proportions were calculated across conditions and developmental stages. Statistical comparisons between genotypes at each time point were performed using Wilcoxon rank-sum tests and quasi-binomial GLMs. Multiple testing correction was applied using the Benjamini–Hochberg procedure. Significance was reported for FDR-adjusted p-values < 0.05. Barplots with standard error and significance annotations were generated for visualization.

### SCENIC analyses

The SCENIC workflow was implemented using pySCENIC (v0.12.1) [34] to evaluate transcription factor activity within both *Gli1*⁺-SuSC and *Axin2*⁺-SuSC populations. Raw count matrices were extracted and converted to loom format. Co-expression modules linking transcription factors (based on hg38 motifs) with candidate target genes were constructed and filtered for motif enrichment. Regulons were defined based on enriched connections, and their activity was quantified per cell using AUCell. Average regulon activity scores were calculated for each SuSC population across developmental stages and genotypes. To identify key regulators, the top six regulons with the highest mean AUCell scores were selected and visually emphasized in transcriptional network plots. These regulons were further assessed for their context-specific activation patterns using Z-score normalization.

### Trajectory analysis of SuSCs

To investigate the lineage dynamics of SuSCs during cranial suture development, we applied the CellRank [32] (v2.0.7) framework in Python. A pseudotemporal ordering of cells was first inferred using the CytoTRACEKernel [31], which estimates differentiation potential based on transcriptional entropy. This kernel was then combined with the RealTimeKernel to model temporal continuity across developmental stages (E14.5, E18.5, and P3). The combined kernel was used to compute a transition matrix representing cell state evolution over time. A coarse-grained fate mapping was derived by fitting the Generalized Perron Cluster Cluster Analysis (GPCCA) model. Terminal states were automatically identified based on the stationary distribution, and lineage probabilities were assigned to each cell. UMAP visualizations were generated to display both differentiation potentials and fate absorption probabilities. Developmental trajectories of SuSCs were interpreted by comparing fate trends across genotypes and time points.

### Monocle3 pseudotime analysis

Monocle3 (v1.4.26) [74] was employed to reconstruct differentiation trajectories without requiring predefined temporal or directional information. Under this unsupervised framework, cells were ordered along a developmental trajectory by leveraging asynchronous variation in their gene expression profiles as a surrogate for progression through the biological process.

### Gene ontology analysis

Gene ontology (GO) analysis was performed separately for upregulated and downregulated differentially expressed genes (DEGs) across developmental timepoints. DEGs were grouped by direction of change, and biological process (BP) terms were enriched using Fisher’s exact test. GO terms with adjusted P-value < 0.05 and more than five associated genes were considered significantly enriched. Top 10 GO terms were selected at each timepoint and visualized by term frequency and significance.

### Gene set variation analysis

Gene set variation analysis (GSVA) was applied to investigate pathway activity shifts across cell clusters and conditions. GSVA scores were computed for each spot using a GO BP term set and were subsequently compared between wild-type and CS samples. Differentially activated pathways were visualized using heatmaps to highlight temporal and genotype-specific changes in biological processes.

### M1 and M2 macrophage polarization scoring

Gene signatures for M1 and M2 macrophage states were curated based on the marker lists published by Martinez et al. [56] Genes overlapping with highly expressed features in macrophage clusters were retained for scoring. Module scores were computed using the AddModuleScore [14] function in Seurat (v5.2.0), with separate scores assigned to M1 and M2 gene sets. A polarization index (M1-M2 Score Difference) was derived by subtracting the M2 module score from the M1 score to evaluate the relative activation of inflammatory versus alternative programs (Figure S4B).

### MultiNicheNet analysis

To explore intercellular communication across cranial suture development, we applied the MultiNicheNet (v2.0.1) R framework to our single-cell dataset. The analysis was performed at each developmental stage (E14.5, E18.5, and P3) using a curated list of sender and receiver cell types. Gene identifiers were standardized to mouse symbols and normalized prior to input. Significant ligand–receptor interactions were predicted with filtering thresholds set as follows: minimum 10 cells per group, log fold change ≥ 0.5, adjusted P-value < 0.05, minimum expression fraction of 5%, and the top 50 ligand-target links retained. Cell–cell interaction networks were constructed by comparing predicted ligand activity across conditions, and downstream visualization highlighted context-specific regulatory signaling during suture development.

### HD spatial sequencing data analysis

After sequencing, the reads were aligned to the Visium Mouse Transcriptome Probe Set (v2.0), and the expression matrix was extracted using the Space Ranger pipeline. Bins with fewer than 10 total counts or genes detected in fewer than 2 cells were removed. Regions of interest (ROIs) were extracted from Loupe Browser (v8.1.2) and used for subsequent spatial data analysis. Gene expression within each ROI was normalized using total-count scaling and log-transformation.

### SpatialCell pipeline for Visium HD analysis

SpatialCell is an open-source pipeline we developed (https://github.com/Xinyan-C/Spatialcell) that integrates Bin2cell [19] segmentation and TopACT [21] classification to enable automated, single-cell–level annotation of Visium HD spatial transcriptomics data.

Nucleus detection was performed on H&E-stained Visium HD tissue sections using QuPath (v0.5.1) [75] with a pre-trained StarDist [20] model (he_heavy_augment), applying 1st–99th percentile normalization, a probability threshold of 0.30, and a pixel size of 0.30 μm. Shape and intensity features for each nucleus were exported as SVG files and converted to NPZ format. Spatial transcriptomics data were loaded with Bin2cell (v0.3.3), filtered to retain genes expressed in ≥ 3 cells and to exclude empty spots, then corrected for image destriping and normalized across spots. Histology-derived labels were mapped into the spatial coordinate system using microns-per-pixel scaling and expanded by a maximum-bin-distance algorithm (2 bins), with k-nearest-neighbor assignment (k = 4) for unlabeled spots. Finally, spots within each cellular territory were aggregated to single-cell resolution by summing gene counts and preserving spatial coordinates for downstream analyses and visualization.

Reference SVM classifiers were trained on single-cell RNA-seq datasets at E14.5, E18.5, and P3 using the TopACT framework (v1.1.0): count matrices and metadata were loaded per time point, and SVM models were fit via the SVCClassifier module with a common gene set standardized across all classifiers. High-resolution Visium HD data(2 μm) were then processed by mapping floating-point spatial coordinates to Bin2cell-derived cellular territories, aligning expression matrices to the training gene set to preserve dimensionality. A cell-constrained neighborhood function was defined to restrict multi-scale analyses within individual cell boundaries: for each query spot, neighbors were drawn only from the same Bin2cell label using Euclidean distance. Results were visualized with Matplotlib scatter plots colored by cell type.

### Niche composition analysis

Visium HD spatial transcriptomics data were loaded and preprocessed with SOAPy [26]. Cell–cell networks were constructed by connecting each cell to neighbors within a 100-pixel Euclidean radius. For each cell, the relative proportions of neighboring cell types were computed in its local network. These composition profiles were clustered (k_max = 20) to define C-niches—unsupervised microenvironmental signatures representing distinct spatial domains.

### Neighborhood analysis

Cell–cell spatial networks were reconstructed using SOAPy [26] with a 40-pixel Euclidean radius to capture direct cellular neighbors. For each sample, one hundred random spatial permutations were generated to establish null distributions of cell type co-occurrence. Observed proximity frequencies were compared against these null distributions to calculate Z-score enrichment, with positive values indicating spatial association and negative values indicating avoidance.

### Spatial gene expression pattern analysis

Marker genes were identified by differential expression with two filter sets: strict (|log₂FC|≥ 1.0, adjusted p < 1 × 10⁻^5^⁰, target ≥ 50% vs. non-target ≤ 50%) and relaxed (|log₂FC|≥ 0.25, adjusted *p* < 0.05). Metagene scores were computed with the Banksy [27] framework by summing functionally related genes into composite signatures and z-score normalizing for cross-sample comparison. Each Visium spot was plotted as a point colored continuously by its metagene score within its Bin2cell-defined territory, preserving single-cell resolution. Linear normalization was applied via Matplotlib’s Normalize class, with symmetric ranges for differential scores and full-range scaling for unidirectional patterns.

To visualize single-gene spatial patterns (Figure S5A), we plotted the log-normalized expression of the indicated gene across spatial coordinates and displayed it as a spatial heatmap. For cross-platform concordance analysis (Figure S5B), we generated cell type–specific marker metagenes in the spatial dataset using the top 5 scRNA-seq marker genes for each scRNA-seq–defined cell type (ranked by log2 fold-change). For each spatial location, the marker metagene score was calculated as the mean expression of these five marker genes (log-normalized values) using the Banksy create_metagene_df function. Metagene scores were visualized as spatial heatmaps. Concordance was assessed by evaluating whether the resulting marker metagene enrichment patterns aligned with expected anatomical regions (e.g., frontal or parietal bone) and known tissue organization.

### Spatial tendency analysis

Spatial tendency analysis was conducted using SOAPy [26] to identify genes exhibiting distance-dependent expression gradients relative to *Gli1*⁺-SuSC regions. *Gli1*⁺-SuSC clusters were binarized into masks with morphological dilation and erosion (kernel size = 35 pixels). For statistical testing, proximal and distal zones were defined by a 50-pixel search radius and a 10-pixel exclusion zone; Wilcoxon rank-sum tests were applied to compare expression between these zones. Spearman correlation coefficients were computed across five concentric distance intervals up to 100 pixels to assess monotonic trends. Polynomial regression models were fitted on external neighborhoods (radius = 100 pixels, sampling fraction = 5%) to detect non-linear patterns. Genes were clustered into ten modules based on their regression trajectories, using a minimum expression-range threshold of 0.03 and FDR-corrected *p* < 0.05.

### Spatial cell–cell communication

Spatial cell–cell communication [26] was inferred by integrating spatial coordinates with normalized gene expression (10,000 counts per cell, log1p-transformed) and a curated mouse ligand–receptor (LR) database compiled via SOAPy [26] from CellChat [76] and Lewis Lab resources. Contact-dependent signaling was modeled by identifying LR pairs within immediate adjacency, capturing membrane-bound and gap-junction interactions. Secretory communication was assessed using a 50-pixel diffusion radius with a distance-weighted influence function assuming isotropic molecular mobility. Permutation testing (100 label shuffles preserving spatial positions) generated null distributions for communication strength and affinity, with significant interactions defined by *p* < 0.05.

### Isolation and culture of suture mesenchymal cells

Following the protocol of Maruyama et al. [77, 78], coronal suture tissue was microdissected from mouse skulls and immediately incubated in DMEM (ThermoFisher, 31600) containing 0.2% collagenase, 10 mM HEPES (Solarbio, H8090), and penicillin–streptomycin (ThermoFisher, 15640055) at 37 °C with gentle agitation (200×*g*) for 60 min. The digest was filtered through a 40 µm nylon strainer and centrifuged (400×*g*, 4 °C, 7 min). Cell pellets were then resuspended in DMEM supplemented with 15% FBS and incubated for downstream assays. Cells were maintained at 37 °C in a humidified atmosphere containing 5% CO₂. The culture medium was changed every 2–3 days. For each biological replicate, suture mesenchymal cells were isolated from pooled coronal sutures of three E18.5 littermates of the same genotype (*Fgfr2*^*C342Y/*+^ or wild type) to obtain sufficient cell numbers for downstream osteogenic induction. Each pooled sample was treated as one biological replicate.

### siRNAs transfection

siRNAs used to knockdown *Foxa3* and negative control were designed and synthesized by OBiO Technology (Shanghai) Corp., Ltd. (listed in Additional file 7). For transfection of siRNAs, Lipofectamine 3000 (ThermoFisher, L3000008) was used according to the manufacturer’s recommendations. siRNA was transfected at a final concentration of 30 nM.

### Quantitative Real-time polymerase chain reaction (RT-qPCR)

Total RNA was isolated from cultured suture mesenchymal cells. Genomic DNA was removed using 5 × gDNA Clean Reaction Mix (2 μl in a 10 μl reaction; 42 °C for 2 min). cDNA was synthesized using the Evo M-MLV reverse transcription system (AG11728; Aikerui) in a 20 μl reaction (37 °C for 15 min, followed by 85 °C for 5 s). Quantitative PCR was performed on a QuantStudio 7 Flex Real-Time PCR System (ABI) using 2 × SG Green qPCR Mix (AG11739; Aikerui). Each 10 μl reaction contained 5 μl 2 × SG Green qPCR Mix, 0.2 μl forward primer (10 μM), 0.2 μl reverse primer (10 μM), 1 μl cDNA, and 3.6 μl nuclease-free water. Relative mRNA expression was normalized to Actb and calculated using the 2^ − ΔΔCt method. Primer sequences are provided in the Additional file 8.

### Osteogenic differentiation, Alizarin Red S staining and ALP staining

For osteogenic differentiation assessment, suture mesenchymal cells were cultured until 70% confluence, then switched to OriCell® Mouse Bone Marrow Mesenchymal Stem Cell Osteogenic Differentiation Medium (MUXMX-90021) with media changes every 3 days. For early osteogenic readout, alkaline phosphatase (ALP) staining was performed after 7 days of induction using an ALP Staining Kit (Beyotime) according to the manufacturer’s instructions. For mineralization assessment, cultures were induced for 14 days and calcium deposition was evaluated by Alizarin Red S staining: cells were fixed with 4% paraformaldehyde, stained with 2% Alizarin Red S solution (pH 4.2) for 20 min, and mineralized nodules were visualized under light microscopy.

### Micro-CT scanning, reconstruction, and visualization

Cranial imaging of mice at P14 and P30 was performed using the Quantum FX micro-CT imaging system (PerkinElmer, USA) at a voxel size of 50 μm. Three-dimensional reconstruction and visualization were performed in 3D Slicer (v5.10.0). Representative 3D views were exported directly from 3D Slicer using a scripted screenshot workflow to ensure consistent visualization settings (e.g., display range/threshold and camera/view orientation) across groups.

### Statistical analysis

Sample sizes and specific parameters are given in the text or figure legends, with measurements being taken from distinct biological replicates. For single-cell RNA sequencing data analysis, cell type proportion comparisons were performed using Wilcoxon rank-sum test and quasi-binomial generalized linear models (GLM) implemented in R. Differential abundance analysis of cell neighborhoods was conducted using the miloR package with graph-overlap weighted FDR correction. Multiple testing correction was applied using the Benjamini–Hochberg false discovery rate (FDR) method as appropriate for each comparison type. Statistical analyses of Alizarin Red S staining quantification were performed using one-way ANOVA followed by Šídák’s multiple comparisons test (GraphPad Prism 10, GraphPad Software Inc, California, USA). Variance homogeneity was verified (Brown-Forsythe test, ns). Data represent four independent biological replicates (n = 4 pools per group); each biological replicate corresponded to pooled suture mesenchymal cells derived from three E18.5 donor animals. Differences were considered statistically significant at *p < 0.05, **p < 0.01, ***p < 0.001 and ****p < 0.0001. Data were presented as mean ± standard error of the mean (SEM).

## Animal ethics

All procedures on mice were performed in adherence with the Guide for the Care and Use of Laboratory Animals (NIH Publication No. 85–23, revised 1996) and institutional/national guidelines. Experiments were approved by the Ethics Committee of the Chinese Academy of Medical Sciences and Peking Union Medical College (Approval No. 2023-A-79; Date of approval: 28 August 2023). Title of the approved project: Endoplasmic reticulum mediated apoptosis of cranial suture mesenchymal stem cells and inflammation in the molecular mechanisms underlying craniosynostosis. All animal experiments conform to the ARRIVE guidelines 2.0. This study did not involve human participants, human material, or human data.

## Consent for publication

Not applicable.

## Competing interests

The authors declare no competing interests.

## Supplementary Information

Additional file 1.

Additional file 2.

Additional file 3.

Additional file 4.

Additional file 5.

Additional file 6.

Additional file 7.

Additional file 8.

Additional file 9.

## Data Availability

The sequenced data generated in this study, including raw and processed files, are available in the GEO repository, accession: GSE303344, GSE303460. The data are currently under embargo and will be publicly available upon publication. Reviewer access can be provided via a reviewer token upon request through the journal’s editorial office. All other data are included in the article and Supplementary Information or available from the corresponding authors upon reasonable request. Code availability

## Abbreviations

SuSC(s): Suture mesenchymal stem cell(s)
CS: Craniosynostosis
WT: Wild type
scRNA-seq: Single-cell RNA sequencing
HD ST-seq: High-definition spatial transcriptomics sequencing (Visium HD)
FFPE: Formalin-fixed paraffin-embedded
H&E: Hematoxylin and eosin
UMI: Unique molecular identifier
ROI: Region of interest
ECM: Extracellular matrix
DEG(s): Differentially expressed gene(s)
GO: Gene Ontology
GSVA: Gene set variation analysis
FDR: False discovery rate
GLM: Generalized linear model
KNN: K-nearest neighbors
SVM: Support vector machine
UMAP: Uniform Manifold Approximation and Projection
t-SNE: t-distributed stochastic neighbor embedding
PFA: Paraformaldehyde
P3: Postnatal day 3
E14.5/E18.5: Embryonic day 14.5 / 18.5

## Code availability

The SpatialCell pipeline for spatial cell identification has been made publicly available on GitHub at: https://github.com/Xinyan-C/Spatialcell. Jupyter notebooks needed to reproduce our analyses (examples/SpatialCell_Demo.ipynb) are included in that repository. A minimal example dataset for E14.5, E18.5, and P3 is archived on Zenodo (https://zenodo.org/records/16400171).
