# Supplementary material for: Spatiotemporal single-cell atlas of suture stem cell dynamics in craniosynostosis
Source: Stem Cell Res Ther. 2026 May 3;17:231. doi: 10.1186/s13287-026-04987-6 (PMC13312657; doi:10.1186/s13287-026-04987-6)
Supplement: Supplementary file 9 — Additional file 9. [file 13287_2026_4987_MOESM9_ESM.pdf]

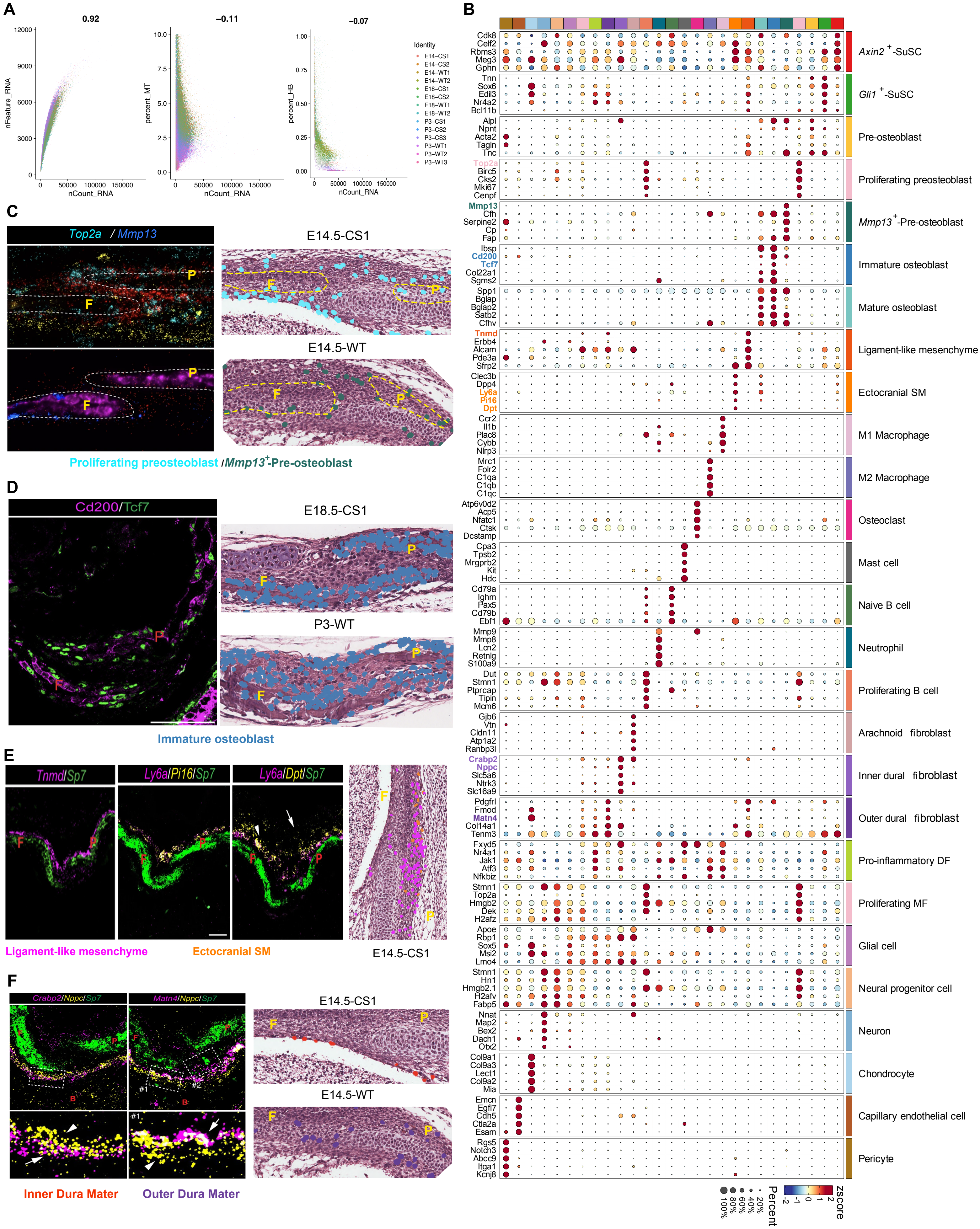
A
B
C
D
E
F
nCount_RNA
nFeature_RNA
percent_MT
percent_HB
Identity
Top2a / Mmp13
E14.5-CS1
E14.5-WT
Proliferating preosteoblast / Mmp13+-Pre-osteoblast
Cd200/Tcf7
E18.5-CS1
P3-WT
Immature osteoblast
Tnmd/Sp7
Ly6a/Pi16/Sp7
Ly6a/Dpt/Sp7
Ligament-like mesenchyme
Ectocranial SM
Crabp2/Nppc/Sp7
Matn4/Nppc/Sp7
Inner Dura Mater
Outer Dura Mater
Axin2+-SuSC
Gli1+-SuSC
Pre-osteoblast
Proliferating preosteoblast
Mmp13+-Pre-osteoblast
Immature osteoblast
Mature osteoblast
Ligament-like mesenchyme
Ectocranial SM
M1 Macrophage
M2 Macrophage
Osteoclast
Mast cell
Naive B cell
Neutrophil
Proliferating B cell
Arachnoid fibroblast
Inner dural fibroblast
Outer dural fibroblast
Pro-inflammatory DF
Proliferating MF
Glial cell
Neural progenitor cell
Neuron
Chondrocyte
Capillary endothelial cell
Pericyte
zscore
Percent

Figure S1. Quality control of scRNA-seq data and validation of SpatialCell cell type annotations (related to Figure 1)

A. Quality control of scRNA-seq data. Scatter plots of RNA counts vs detected genes, mitochondrial and hemoglobin gene percentages, colored by sample.

B. Dot plot showing the expression of markers for each cell type. Colored markers represent examples of genes with expression patterns consistent with previous in situ hybridization studies and validated by spatial transcriptomics analysis in this study. SM, Suture mesenchyme cell; DF, dural fibroblast; MF, meningea fibroblast.

C-F. Validation of SpatialCell cell type annotations using published in situ hybridization patterns. F, frontal bone; P, parietal bone.

(C) Proliferating pre-osteoblast markers *Top2a* and *Mmp13* (reproduced under terms of the CC-BY license[11]. Copyright © 2021, Holmes et al., published by Springer Nature) compared with SpatialCell annotations on H&E staining (this study).

(D) Immature osteoblast markers *Cd200*/*Tcf7* (reproduced under terms of the CC-BY license[15]. Copyright © 2021, Farmer et al., published by Springer Nature) compared with SpatialCell annotations on H&E staining (this study).

(E) Ligament-like mesenchyme marker *Tnmd* and ectocranial suture mesenchyme markers *Ly6a*, *Pi16* and *Dpt* (reproduced under terms of the CC-BY license[15]. Copyright © 2021, Farmer et al., published by Springer Nature) compared with SpatialCell annotations on H&E staining (this study).

(F) Inner and outer dura mater markers *Crabp2*/*Nppc* and *Matn4* (reproduced under terms of the CC-BY license[15]. Copyright © 2021, Farmer et al., published by Springer Nature) compared with SpatialCell annotations on H&E staining (this study).

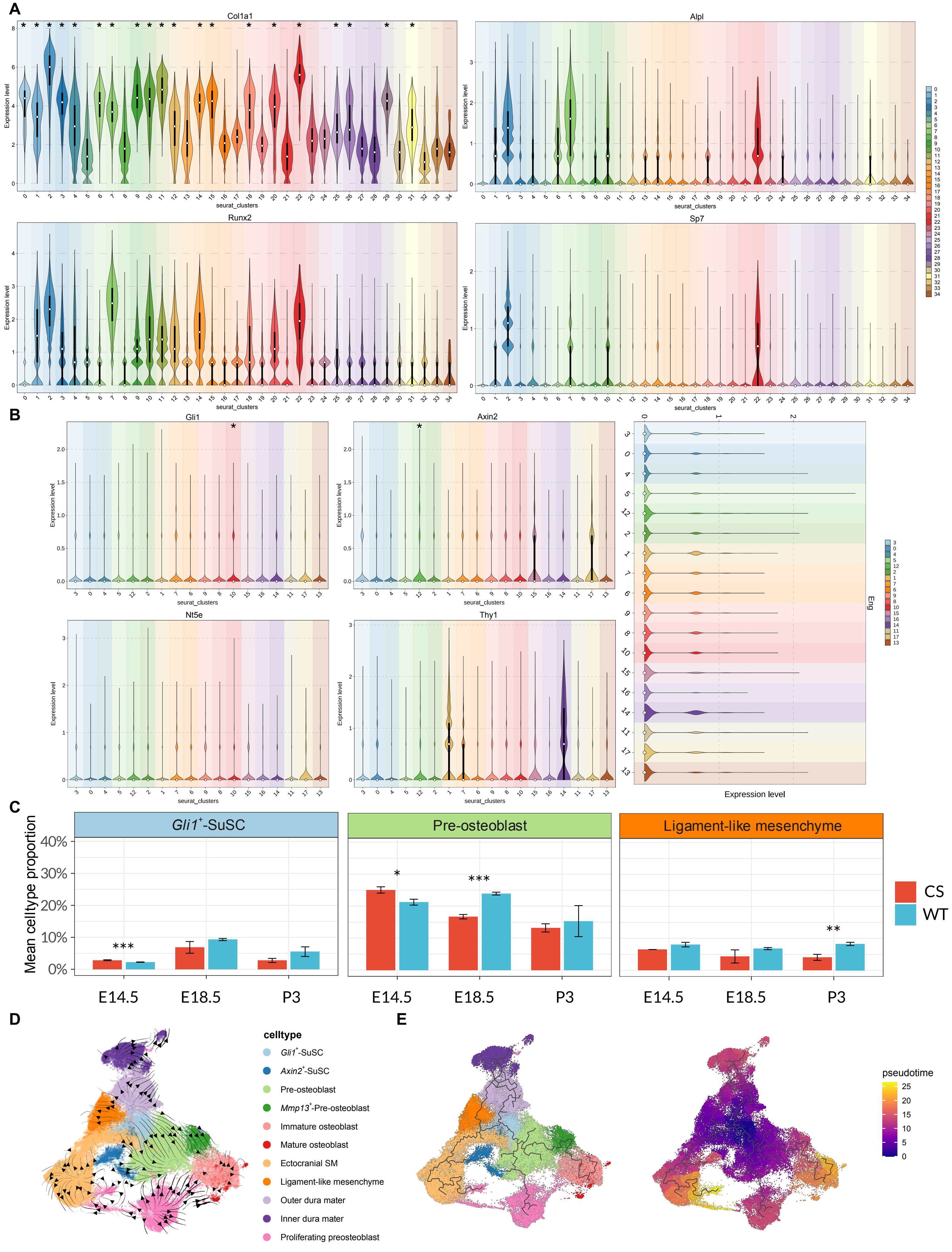

Figure S2. scRNA-seq analysis reveals SuSC heterogeneity and osteogenic differentiation trajectories (related to Figure 3)

A. scRNA-seq identification of suture mesenchymal cells. Violin plots of marker genes (Col1a1, Alpl, Runx2, Sp7) with selected populations marked by asterisks.

B. scRNA-seq identification of SuSCs. Violin plots of marker genes (Gli1, Axin2, Nt5e, Thy1, Eng) with selected populations marked by asterisks.

C. Cell-type proportions (±SE) comparing CS vs WT at each timepoint with FDR-adjusted significance: *q < 0.05; **q < 0.01; ***q < 0.001.

D-E. Differentiation trajectories along the SuSC-to-osteoblast axis of WT samples by CytoTRACE (D) and Monocle3 pseudotime analysis (E).

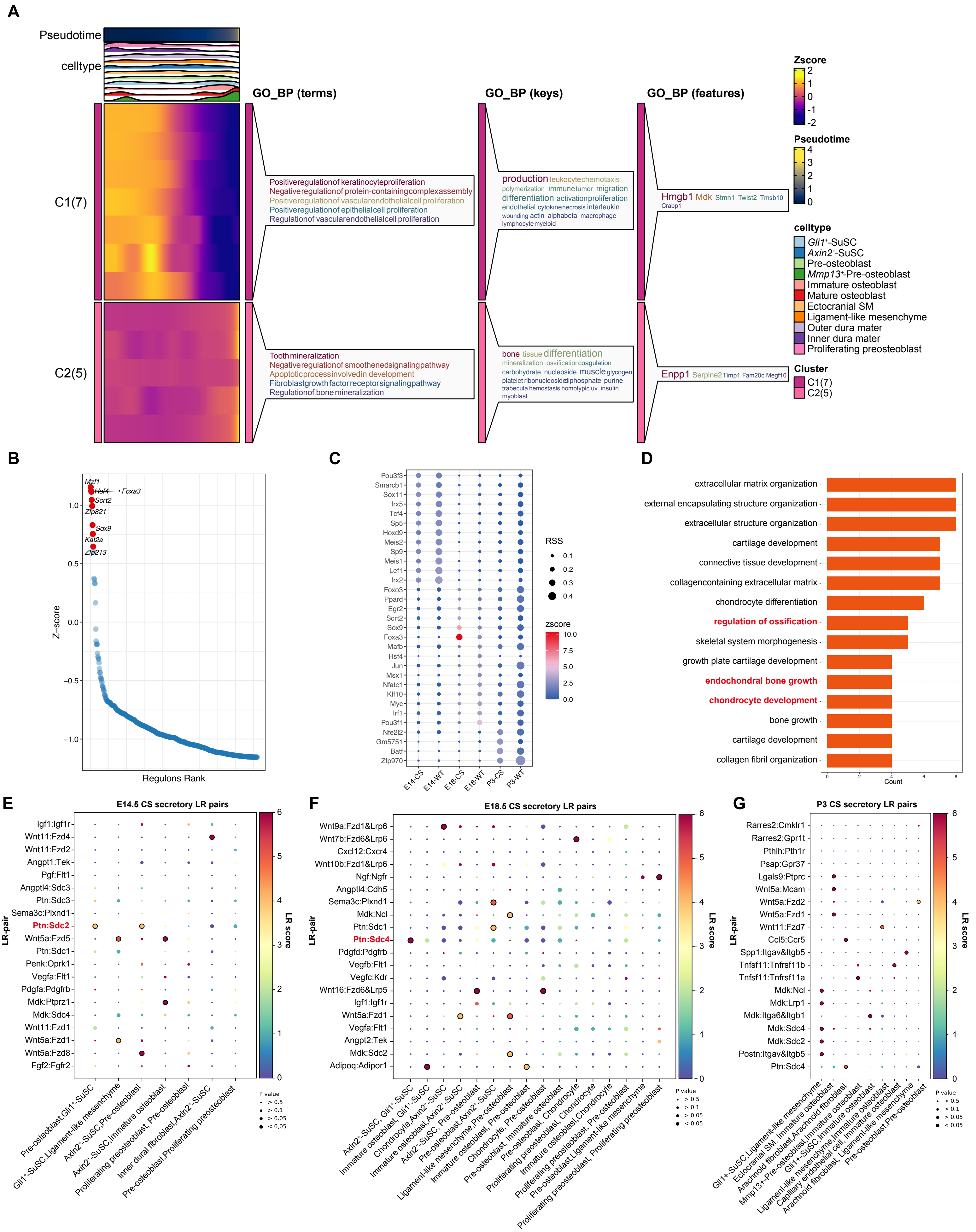

Figure S3. Analysis of transcriptional dynamics and cell communication in SuSC differentiation(related to Figure 4)

A. Heatmap of WT dynamic gene expression along SuSC-to-osteoblast pseudotime trajectory. Two temporal clusters with associated GO biological process terms are shown.

B. Scatter plots of top 6 TF regulons based on Z scores in *Axin2*+-SuSCs.

C. Heatmap of regulon specificity scores (RSS) across developmental timepoints and disease conditions in *Axin2*+-SuSCs.

D. Bar plot of GO biological process enrichment for Foxa3 target genes — highlighting ossification and endochondral bone growth processes.

E-G. Dot plots showing spatial secretory cell-cell communication patterns across developmental timepoints in CS samples. Significant ligand-receptor pairs are highlighted with circles. LR, ligand-receptor.

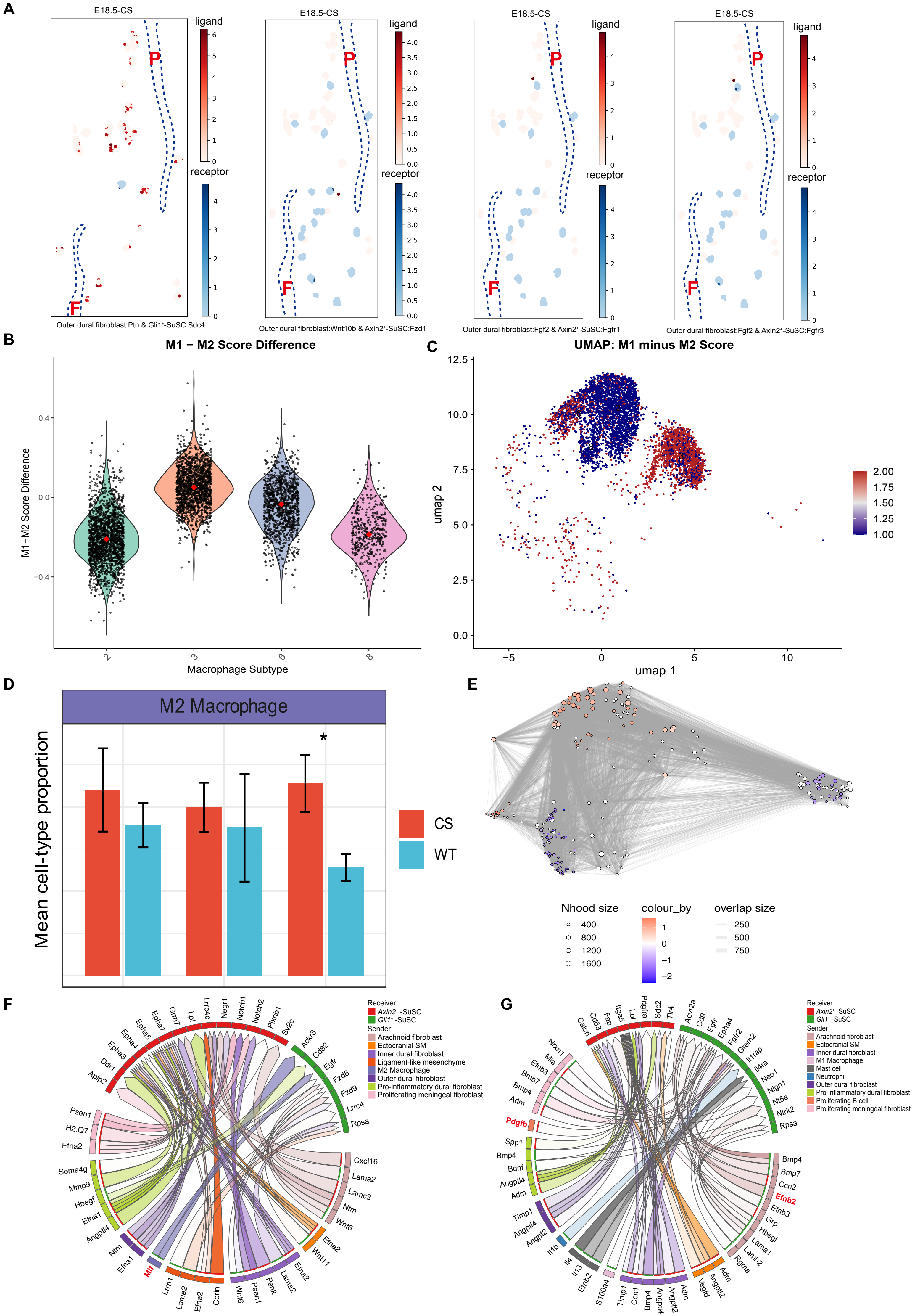
A
E18.5-CS
ligand
receptor
P
F
Outer dural fibroblast:Ptn & Gli1⁺-SuSC:Sdc4
Outer dural fibroblast:Wnt10b & Axin2⁺-SuSC:Fzd1
Outer dural fibroblast:Fgf2 & Axin2⁺-SuSC:Fgfr1
Outer dural fibroblast:Fgf2 & Axin2⁺-SuSC:Fgfr3
B
M1 − M2 Score Difference
M1−M2 Score Difference
Macrophage Subtype
C
UMAP: M1 minus M2 Score
umap 1
umap 2
D
M2 Macrophage
Mean cell-type proportion
CS
WT
*
E
Nhood size
400
800
1200
1600
colour_by
overlap size
250
500
750
F
Receiver
Axin2⁺ -SuSC
Gli1⁺ -SuSC
Sender
Arachnoid fibroblast
Ectocranial SM
Inner dural fibroblast
Ligament-like mesenchyme
M2 Macrophage
Outer dural fibroblast
Pro-inflammatory dural fibroblast
Proliferating meningeal fibroblast
Mif
G
Receiver
Axin2⁺ -SuSC
Gli1⁺ -SuSC
Sender
Arachnoid fibroblast
Ectocranial SM
Inner dural fibroblast
M1 Macrophage
Mast cell
Neutrophil
Outer dural fibroblast
Pro-inflammatory dural fibroblast
Proliferating B cell
Proliferating meningeal fibroblast
Pdgfb
Efnb2

Figure S4. Cell–cell communication, macrophage polarization, and cellular abundance dynamics in craniosynostosis (related to Figures 5 and 6)

A. Spatial cell communication analysis in E18.5 CS, showing outer dural fibroblast and SuSCs Ligand-receptor spatial secretory communication.

Color intensity indicates normalized expression level. F, frontal bone; P, parietal bone.

B-C. Macrophage M1–M2 polarization across selected clusters:

(B) Violin plot showing the distribution of the polarization index (M1 – M2 Score difference) in macrophage clusters 2, 3, 6 and 8.

(C) UMAP embedding of the same macrophage clusters, with each cell colored according to its M1 – M2 score difference.

D. Cell-type proportions (±SE) comparing CS vs WT at each timepoint with FDR-adjusted significance: *q < 0.05.

E. Milo analysis UMAP illustrating differences in M2 macrophage abundance between P3 CS and WT. Each node represents a neighborhood—its size is proportional to the number of cells in that neighborhood, and the edge width reflects the number of overlapping cells between neighborhoods. Nodes are colored according to the log2 fold change in cell abundance between the two conditions.

F-G. Circos plot of top 50 cell-cell interactions between cell types (ligand) and SuSCs (receptor) in E14.5 (left) and P3 (right).

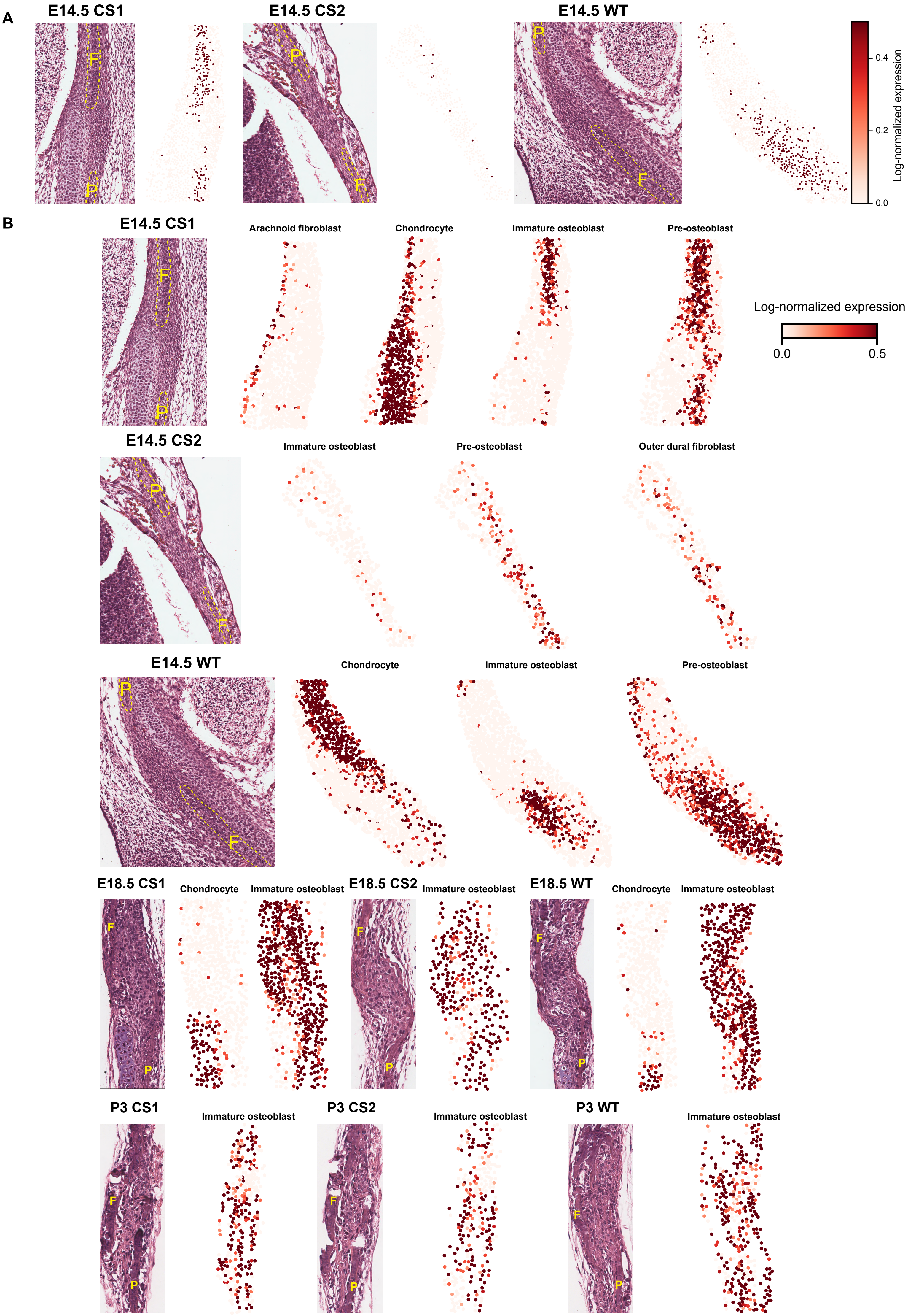

A
E14.5 CS1
E14.5 CS2
E14.5 WT
Log-normalized expression
0.0
0.2
0.4
B
E14.5 CS1
Arachnoid fibroblast
Chondrocyte
Immature osteoblast
Pre-osteoblast
Log-normalized expression
0.0
0.5
E14.5 CS2
Immature osteoblast
Pre-osteoblast
Outer dural fibroblast
E14.5 WT
Chondrocyte
Immature osteoblast
Pre-osteoblast
E18.5 CS1
Chondrocyte
Immature osteoblast
E18.5 CS2
Immature osteoblast
E18.5 WT
Chondrocyte
Immature osteoblast
P3 CS1
Immature osteoblast
P3 CS2
Immature osteoblast
P3 WT
Immature osteoblast

Figure S5. Spatial validation of bone-region mapping and scRNA-seq–derived cell-type signatures

A. Spatial distribution of Sp7 expression in E14.5 samples. Spatial heatmap showing normalized *Sp7* expression. Color intensity represents log-normalized expression. (F, frontal bone; P, parietal bone)

B. Spatial validation of scRNA-seq-derived cell type markers. Spatial heatmaps of cell type-specific metagene expression derived from scRNA-seq marker genes. Metagene expression represent the averaged log-normalized expression of top 5 marker genes for each cell type identified from scRNA-seq analysis.

Color intensity indicates metagene expression level. Spatial concordance between scRNA-seq-derived markers and known anatomical locations validates the cross-platform reliability of cell type identification. (F, frontal bone; P, parietal bone)

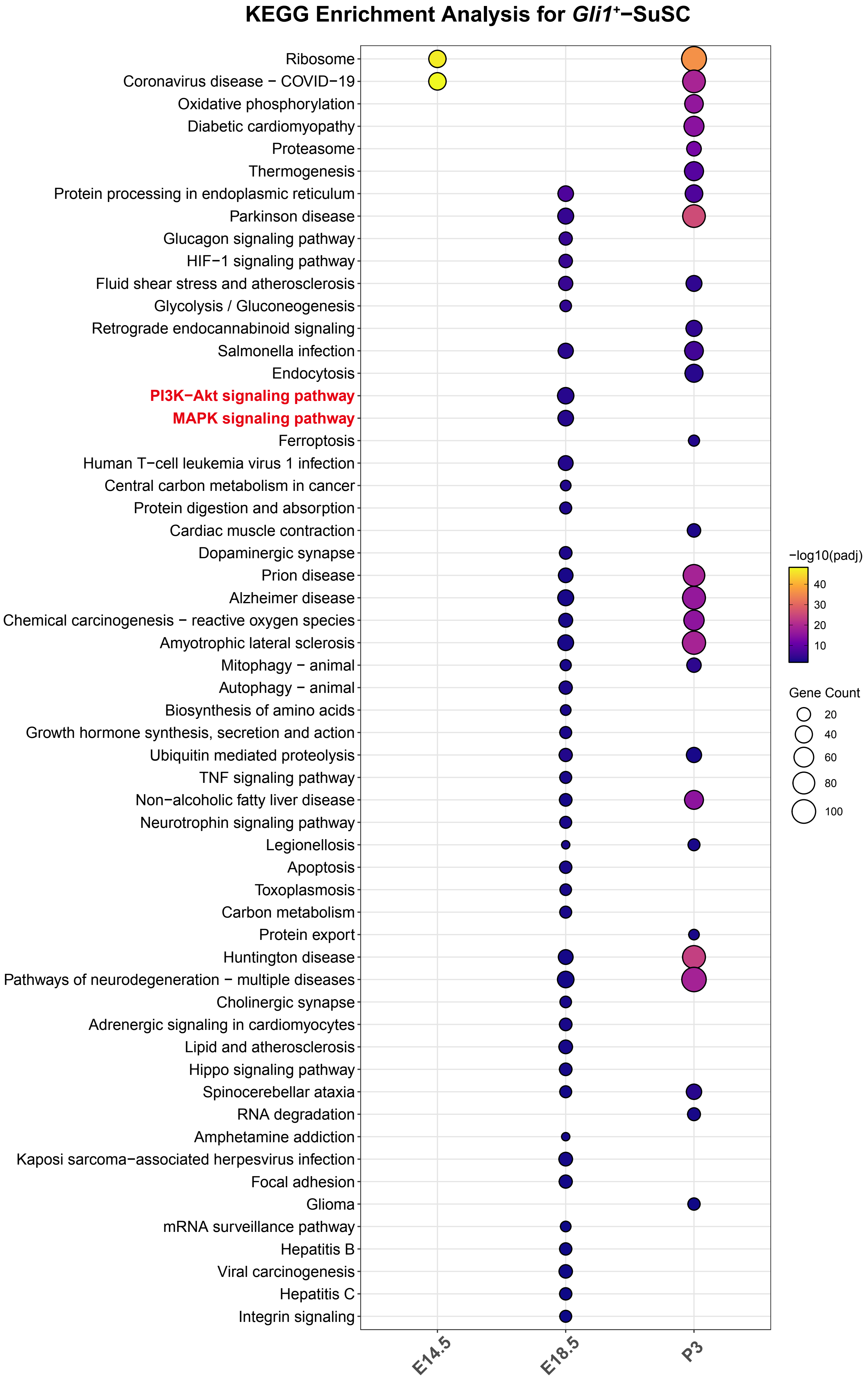

KEGG Enrichment Analysis for Gli1⁺−SuSC
Ribosome
Coronavirus disease − COVID−19
Oxidative phosphorylation
Diabetic cardiomyopathy
Proteasome
Thermogenesis
Protein processing in endoplasmic reticulum
Parkinson disease
Glucagon signaling pathway
HIF−1 signaling pathway
Fluid shear stress and atherosclerosis
Glycolysis / Gluconeogenesis
Retrograde endocannabinoid signaling
Salmonella infection
Endocytosis
PI3K−Akt signaling pathway
MAPK signaling pathway
Ferroptosis
Human T−cell leukemia virus 1 infection
Central carbon metabolism in cancer
Protein digestion and absorption
Cardiac muscle contraction
Dopaminergic synapse
Prion disease
Alzheimer disease
Chemical carcinogenesis − reactive oxygen species
Amyotrophic lateral sclerosis
Mitophagy − animal
Autophagy − animal
Biosynthesis of amino acids
Growth hormone synthesis, secretion and action
Ubiquitin mediated proteolysis
TNF signaling pathway
Non−alcoholic fatty liver disease
Neurotrophin signaling pathway
Legionellosis
Apoptosis
Toxoplasmosis
Carbon metabolism
Protein export
Huntington disease
Pathways of neurodegeneration − multiple diseases
Cholinergic synapse
Adrenergic signaling in cardiomyocytes
Lipid and atherosclerosis
Hippo signaling pathway
Spinocerebellar ataxia
RNA degradation
Amphetamine addiction
Kaposi sarcoma−associated herpesvirus infection
Focal adhesion
Glioma
mRNA surveillance pathway
Hepatitis B
Viral carcinogenesis
Hepatitis C
Integrin signaling
E14.5
E18.5
P3
−log10(padj)
40
30
20
10
Gene Count
20
40
60
80
100

Figure S6. KEGG pathway enrichment of CS-upregulated genes in *Gli1*$^+$ SuSCs (CS vs WT across stages)

KEGG pathway enrichment analysis of genes upregulated in CS versus WT within *Gli1*$^+$ SuSCs across developmental stages. Each dot represents a KEGG pathway. The x-axis indicates genotype comparisons by stage (E14.5, E18.5, P3; CS vs WT), and the y-axis lists enriched pathways. Dot size reflects the number of genes contributing to each pathway (Count), and dot color indicates enrichment significance (−log10 adjusted P value, FDR-corrected).

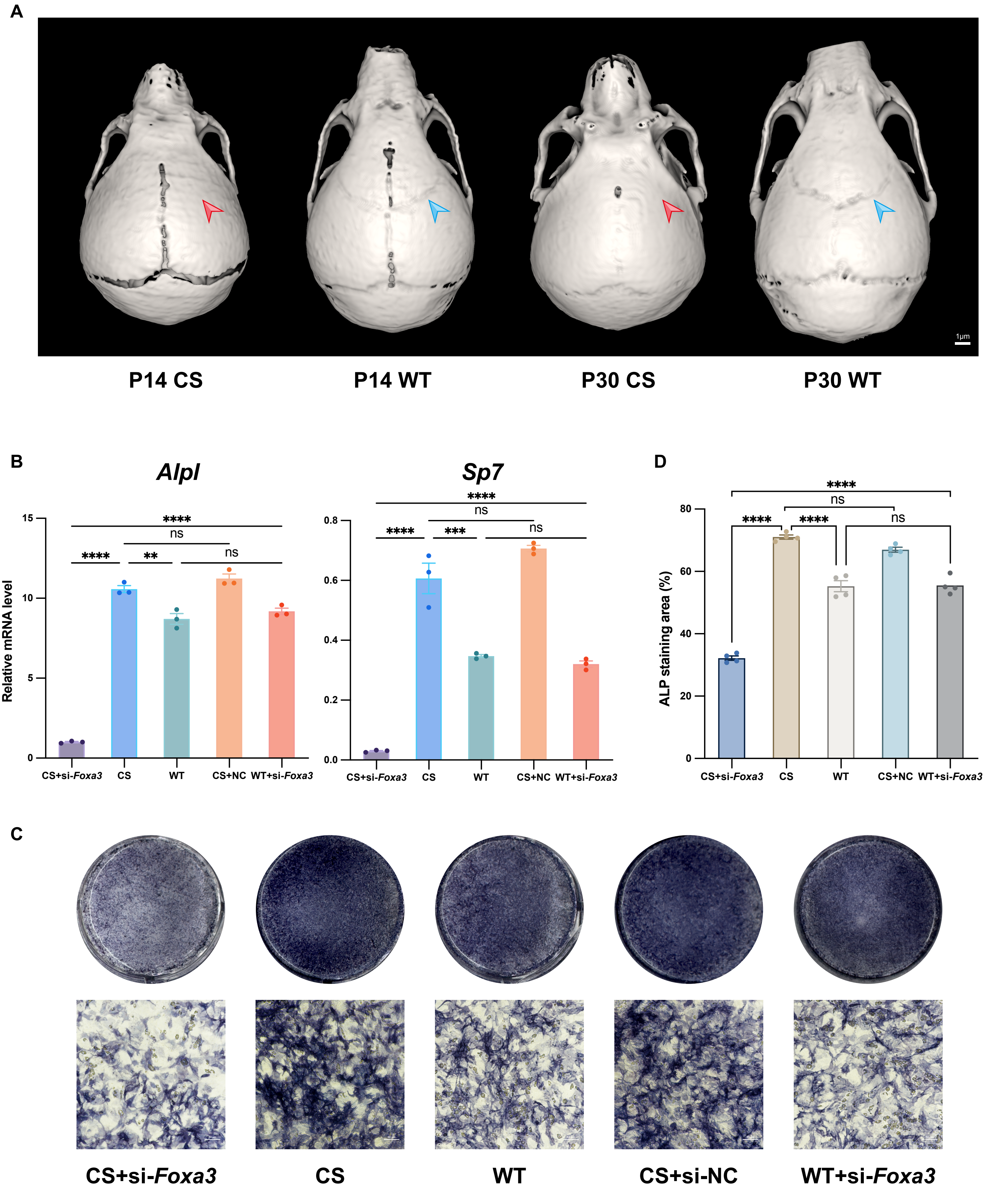

Figure S7. Additional validation of model phenotype and *Foxa3* perturbation outcomes

A. Micro-CT images of skulls from *Fgfr2*$^{C342Y/+}$ (craniosynostosis, CS) and wild-type (WT) mice at P14 and P30. Red arrowheads indicate coronal suture fusion in CS mice, whereas blue arrowheads indicate a patent coronal suture in WT mice. Scale bar, 1 mm. n = 5 mice per group.

B. RT-qPCR analysis of osteogenic marker gene expression (*Alpl* and *Sp7*) in E18.5 coronal suture mesenchymal cultures from CS and WT mice with *Foxa3* silencing under osteogenic induction (day 7). Relative mRNA levels were normalized to Actb. n = 4 per group. Significance was determined by one-way ANOVA, **p < 0.01, ***p < 0.001, and ****p < 0.0001.

C–D. ALP staining (C) and quantification of Alkaline phosphatase (ALP) positive area (%) (D) in E18.5 coronal suture mesenchyme cultures from CS and WT mice with *Foxa3* silencing under osteogenic induction (day 7). n = 4 per group. Significance was determined by one-way ANOVA, **p < 0.01, ***p < 0.001, and ****p < 0.0001.
